# The dark side of pseudoscorpion diversity: The German Barcode of Life campaign reveals high levels of undocumented diversity in European false scorpions

**DOI:** 10.1002/ece3.8088

**Published:** 2021-09-08

**Authors:** Christoph Muster, Jörg Spelda, Björn Rulik, Jana Thormann, Laura von der Mark, Jonas J. Astrin

**Affiliations:** ^1^ Zoologisches Institut und Museum Universität Greifswald Greifswald Germany; ^2^ SNSB‐Zoologische Staatssammlung München Munich Germany; ^3^ Zoologisches Forschungsmuseum A. Koenig ZFMK Bonn Germany

**Keywords:** BIN divergence, DNA barcode library, false scorpions, morphological crypsis, *Neobisium carcinoides*, species delimitation

## Abstract

DNA barcoding is particularly useful for identification and species delimitation in taxa with conserved morphology. Pseudoscorpions are arachnids with high prevalence of morphological crypsis. Here, we present the first comprehensive DNA barcode library for Central European Pseudoscorpiones, covering 70% of the German pseudoscorpion fauna (35 out of 50 species). For 21 species, we provide the first publicly available COI barcodes, including the rare *Anthrenochernes stellae* Lohmander, a species protected by the FFH Habitats Directive. The pattern of intraspecific COI variation and interspecific COI variation (i.e., presence of a barcode gap) generally allows application of the DNA barcoding approach, but revision of current taxonomic designations is indicated in several taxa. Sequences of 36 morphospecies were assigned to 74 BINs (barcode index numbers). This unusually high number of intraspecific BINs can be explained by the presence of overlooked cryptic species and by the accelerated substitution rate in the mitochondrial genome of pseudoscorpions, as known from previous studies. Therefore, BINs may not be an appropriate proxy for species numbers in pseudoscorpions, while partitions built with the ASAP algorithm (Assemble Species by Automatic Partitioning) correspond well with putative species. ASAP delineated 51 taxonomic units from our data, an increase of 42% compared with the present taxonomy. The *Neobisium carcionoides* complex, currently considered a polymorphic species, represents an outstanding example of cryptic diversity: 154 sequences from our dataset were allocated to 23 BINs and 12 ASAP units.

## Graphical abstract

A first extensive COI barcode library for European pseudoscorpions, covering 70% of the German fauna, revealed unexpected high levels of cryptic diversity. BIN and ASAP partitions provide an interim taxonomic system on which thorough taxonomic revisions of morphologically uniform species groups can be based.

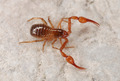

## 1 INTRODUCTION

DNA barcoding has greatly contributed to the inventory of global biodiversity. Since establishment of the BOLD database (Ratnasingham & Hebert, 2007), more than 9 million DNA barcodes from >320,000 species have been generated (https://www.boldsystems.org/index.php/TaxBrowser_Home; accessed 22 January 2021). Originally designed as a tool to facilitate species identification (Hebert et al., 2003), DNA barcoding has also revealed cryptic diversity across the animal kingdom (Hebert et al., 2004; Ramirez et al., 2017; Reier et al., 2020; Witt et al., 2006). The large depositories of genetic data enclose a growing number of “dark taxa,” that is, sequences without reference to scientific species names, as originally described by Page (2016). Dark taxa may contain datasets of species that could not be allocated to existing Linnean names, either due to lack of taxonomic expertise or because they represent undescribed species (the latter use is becoming more widespread, e.g., Ryberg & Nilsson, 2018). The study of dark taxa is the main focus of some recent barcoding campaigns, for example, in the 3rd phase of the German Barcode of Life Initiative GBOL (https://bolgermany.de/home/gbol3/). A taxon with a putative high proportion of dark taxa that have received little attention in the past is the arachnid order Pseudoscorpiones. In the BOLD database, almost half of the pseudoscorpion sequences are not specified at the species level (https://www.boldsystems.org/index.php/Public_SearchTerms?query=Pseudoscorpiones, accessed 22 January 2021).

Compared to the large arachnid orders, pseudoscorpions are less intensely studied phylogenetically (Benavides et al., 2019), taxonomically (Cameron & Buddle, 2019), or with respect to habitat requirements and niche occupation (Battirola et al., 2017). In consequence, although ubiquitous and of functional significance in soil biota, pseudoscorpions are still rarely used in applied environmental studies (Gerlach et al., 2013). Also, the molecular record is limited. For example, a survey of the nucleotide database at GenBank (https://www.ncbi.nlm.nih.gov/nuccore/; accessed 14 January 2021) resulted in 2,303 hits for “Pseudoscorpiones,” which is roughly 0.6 items per species, while “Araneae” yielded 1,290,065 results (~26 items/species) and “Opiliones” 16,200 matches (~2.4 per species). Similarly, in the public data portal of BOLD (https://www.boldsystems.org; accessed 14 January 2021) 1845 sequences of pseudoscorpions were found, representing 295 species with barcodes (on average 0.5 sequences per species, species coverage ~8%). For spiders, 126,753 COI barcodes have been generated from 7,350 species (2.6 sequences/species, species coverage ~15%), and for harvestmen, 8,357 barcodes have been generated from 971 species (1.2 sequences/species, species coverage ~14.5%).

The Central European pseudoscorpions roughly divide into two ecological guilds (Legg, 1975; Weygoldt, 1966). About one‐half of the species are soil‐dwelling inhabitants which can be found in leaf litter and under stones. Most of these species prefer habitats of high moisture. Leaf litter is considered the ancestral habitat of pseudoscorpions (Bell et al., 1999), and thus, it is not surprising that this guild is composed by species of ancestral lineages, in particular representatives of Chthonioidea and Neobisioidea (sensu Benavides et al., 2019). Their dispersal capacity is very low, and therefore, they show tendencies of remarkable patchy distribution at local and regional scales (Buddle, 2005) and short‐range endemism at the biogeographical scale (Opatova & Šťáhlavský, 2018). The other half of species inhabits structures that are intrinsically patchy and temporary, such as tree hollows, rotten tree bark, animal nests, and feces. Most pseudoscorpions of this guild belong to the Cheliferoidea, most numerous to Chernetidae. In order to be able to colonize such isolated and transitional habitats, they developed phoresy as an effective mode of passive transport (Červená et al., 2019; Poinar et al., 1998). Phoresy is an interaction in which nonvagile animals attach themselves onto mobile host species for the purpose of dispersal (White et al., 2017). Generally, phoretic pseudoscorpions have larger distribution ranges than species not showing phoretic behavior (Legg, 1975). In addition, many phoretic species were able to synanthropically colonize human‐made structures as secondary habitats, such as barns, attics, and compost heaps (Ressl, 1983).

It is likely that the strikingly different dispersal capacities between phoretic and nonphoretic pseudoscorpions do not only affect range sizes, but also affect the genetic structure within species. In nonvagile species, a pattern of deep phylogeographic structure with mostly allopatric lineages should dominate, while in vagile species shallow gene trees with overlapping lineages are anticipated (Avise, 2000). Highly structured species with evident correlation between genetic and geographic distances have indeed been found in Australian Chthonioidea (Harms, 2018; Harrison et al., 2014). On the other hand, phylogeographic studies in phoretic pseudoscorpions recorded a lack of geographic structure in haplotype networks, with haplotypes shared among localities that are up to hundreds of kilometers apart (Harvey et al., 2015; Opatova & Šťáhlavský, 2018). However, comprehensive data are largely missing. Small barcode libraries have solely been published for Canadian (Cameron & Buddle, 2019) and South Korean pseudoscorpions (Ohira et al., 2018).

Here, we release a DNA barcode reference library for Central European pseudoscorpions that has been generated in the first and second phases of the GBOL project. German Barcode of Life started in 2012. It is one of the largest national barcoding initiatives and aims to generate DNA barcodes for all animal species in Germany (Geiger, Astrin, et al., 2016). Results have been published for various groups, including Araneae and Opiliones (Astrin et al., 2016), Myriapoda (Wesener et al., 2016), Coleoptera (Hendrich et al., 2015; Rulik et al., 2017), Heteroptera (Havemann et al., 2018), Hymenoptera (Schmid‐Egger et al., 2019; Schmidt et al., 2015, 2017), and Diptera (Morinière et al., 2019). The results for pseudoscorpions confirm high levels of unresolved taxonomy in Germany, a region with a long history of faunistic and taxonomic researches. We present an almost unrivaled example of extraordinarily high levels of cryptic diversity within a presumed “polymorphic species.” We are convinced that our results will stimulate consequential taxonomic revisions of several species complexes among the European pseudoscorpions.

## 2 MATERIAL AND METHODS

### 2.1 Sampling

**FIGURE 1:**
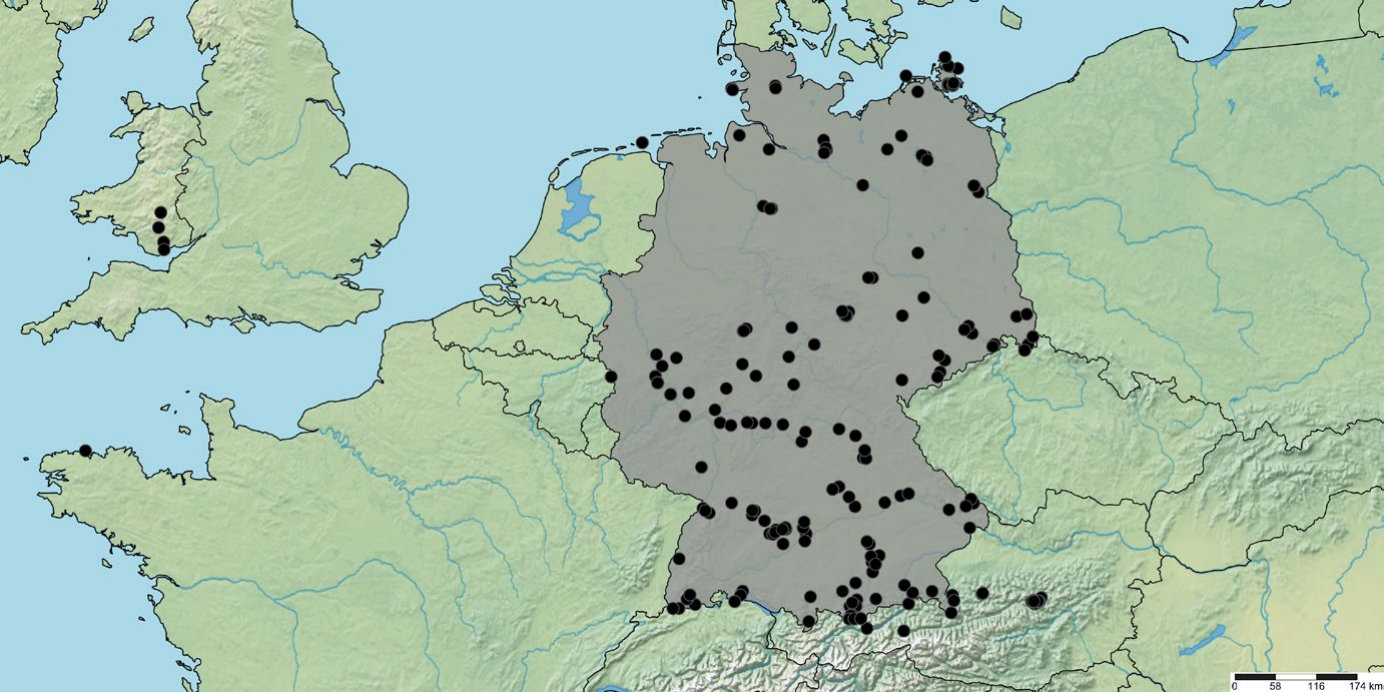
Sampling localities of pseudoscorpions in Germany and adjacent countries in course of the GBOL project. The map was produced with SimpleMappr (www.simplemappr.net)

The released dataset contains sequences of 459 specimens (Appendix S1). In accordance with the objectives of GBOL, most specimens were collected in Germany (421 specimens, 92%). Further material originates from Austria (28 specimens, 6%), from UK (Wales, 7 specimens), and single specimens from France, Switzerland, and Slovenia. Within Germany, strong geographic sampling bias is not evident (Figure 1).

Two GBOL institutions contributed to the processing of pseudoscorpion data: Zoologisches Forschungsmuseum Alexander Koenig in Bonn (ZFMK, 266 specimens with barcode), and Zoologische Staatssammlung München (ZSM, 193 specimens with barcode). Voucher specimens are stored in these institutions. A network of associated researchers contributed to the collection of pseudoscorpions. Most specimens were collected by Jörg Spelda (132) and Christoph Muster (112). The majority of specimens were collected by hand or by sieving of litter and wood mold and were immediately transferred into pure ethanol. A significant proportion (91 specimens) originates from malaise traps that were installed in the course of the Global Malaise Trap Program from 2009 to 2016 (Geiger, Moriniere, et al., 2016). Sampling efforts were increased for some species that showed signals of cryptic diversity in preliminary studies. Material was collected in compliance with national and international laws, regulations, and conventions. Fieldwork permits for protected areas were issued by the responsible state environmental agencies.

Pseudoscorpions were determined to species level using the keys of Christophoryová et al. (2011), De Vore‐Scribante (1999), Legg and Farr‐Cox (2016), and Mahnert (2004), which reflect latest taxonomic knowledge except for some Chthoniidae, for which we followed Gardini (2013, 2014) and Zaragoza (2017). Nomenclature follows the current checklist of pseudoscorpiones of Germany (Muster & Blick, 2016) and Zaragoza (2017). To represent the status quo of pseudoscorpion taxonomy, we assigned Linnean names according to these sources, even in cases of obvious disagreement with our molecular data. In our opinion, taxonomic and nomenclatorial consequences shall reside with the authors of subsequent revisions, preferably following an integrative approach. However, specimens with conspicuous placement in preliminary NJ trees were carefully rechecked morphologically and corrected in cases of misidentification.

Sequence data are available in the public data set “DS‐GBPSS GBOL‐Pseudoscorpiones Germany” (http://doi.org/10.5883/DS‐GBPSS ) on BOLD and on GenBank (accs‐no MN621854‐MN621856, MW995987‐MW996442, also available in Appendix S1). Alignment of the sequences is placed in Appendix S4.

### 2.2 Laboratory procedures

Analyses were performed at ZFMK and—for ZSM samples—at the Canadian Centre for DNA Barcoding (CCDB) in Guelph. Total genomic DNA was isolated from legs (ZSM) or nondestructively from complete specimens (ZFMK) to preserve undamaged vouchers. Entire specimens were incubated in 180µl Qiagen ATL tissue lysis buffer at 56°C for 16 hr by addition of 20µl Qiagen proteinase K. The following laboratory workflow was implemented at ZFMK: A Qiagen (Hilden, Germany) BioSprint96 magnetic bead extractor and corresponding kits were used, strictly following the manufacturer's specifications. We amplified 658 bp from the 5'‐end of the COI (cytochrome c oxidase subunit I) gene with primers HCO2198‐JJ and LCO1490‐JJ (Astrin & Stüben, 2008) or alternatively HCO2198‐JJ2 and LCO1490‐JJ2 (Astrin et al., 2016) using standard PCR conditions (see Astrin et al., 2016) in reaction volumes of 20 μl, including 2.0 μl of DNA template, and using the “Multiplex PCR Master Mix” (Qiagen). PCR products were subsequently sent for bidirectional Sanger sequencing to BGI (Hong Kong, China). CCDB laboratory protocols are available under https://ccdb.ca/resources/.

### 2.3 Data analysis

DNA sequences were assembled, inspected, and aligned using Geneious vers. R7 (Biomatters, Auckland, New Zealand). Alignment length was set to 658 bp. Sequences shorter than 500 bp were excluded from distance and species delimitation analyses to avoid artifacts. The Perl script DiStats (Astrin et al., 2016) was used to statistically explore the genetic distances in the dataset (intraspecific distances, closest species pairs). Neighbor joining trees were built in Geneious. Maximum‐likelihood (ML) reconstructions used RAxML‐HPC vers. 8.2.12 (Stamatakis, 2014). For the ML searches, a GTR + Γ model of sequence evolution was applied following the program recommendations. The dataset was partitioned to treat 3rd codon positions separately from 1st and 2nd positions. The analysis used the “‐f a” option (bootstrap analysis and search for best‐scoring ML tree in one program run) and included 10,000 bootstrap replicates. We chose a camel spider, a scorpion, and a mite sequence from BOLD as out‐groups.

BINs (Barcode Index Numbers) were assigned and registered by the refined single linkage (RESL) algorithm that runs weekly on BOLD (Ratnasingham & Hebert, 2013). The BIN system provides operational taxonomic units (OTUs) that can be used to compare the concordance between barcode sequence clusters and a priori taxonomic designations. A major advantage of the BIN system is the comparability across taxa. For many groups, a close correspondence of BINs and Linnean species has been observed (Hausmann et al., 2013; Hendrich et al., 2015; Schmidt et al., 2015), and thus, BIN counts are sometimes used as a proxy for species richness (Hebert et al., 2016). However, in situations of significant population structure within species, for example, caused by complex biogeographic histories, BIN and other single‐locus delineation methods tend to oversplitting by mistaking population‐level lineages as putative species (Kekkonen & Hebert, 2014; Muster & Michalik, 2020; Sukumaran & Knowles, 2017). Accelerated mutation rates, as uncovered in pseudoscorpions (Arabi et al., 2012), may also result in overestimation of species diversity if the method is based on a universal upper threshold for intraspecific distance (e.g., 2.2% as assumed in BIN assignment on BOLD). We therefore used the ASAP procedure (assemble species by automatic partitioning; Puillandre et al., 2021) for the delineation of candidate species in our pseudoscorpion dataset. ASAP is a recent advancement of the automatic barcode gap discovery ABGD (Puillandre et al., 2012). Among the many molecular species delimitation methods (Rannala & Yang, 2020), the ABGD delineation often generated the most conservative estimates in terms of species numbers and the best concordance with morphospecies (Pentinsaari et al., 2017; Zhou et al., 2019). A major improvement of ASAP as compared to ABGD is that it does not require the a priori definition of a distance threshold. ASAP is based on a hierarchical clustering algorithm which successively merges sequences into groups using ranked pairwise distances. For each new partition, ASAP computes the probability of panmixia (*p*‐value) and the relative barcode gap width (W). The ASAP score is the average of the ranks of both metrics, the lower the score the better the partition. Thus, the method infers the distance threshold for species delineation from the data and therefore can take accelerated speciation rates into account. We used the ASAP web interface (https://bioinfo.mnhn.fr/abi/public/asap/asapweb.html, accessed 29 January 2021) for delineation using p‐distances and the default settings.

## 3 RESULTS

Our dataset contains DNA barcodes of 36 pseudoscorpion species, of which 35 occur in Germany (Table 1). The latest checklist of Pseudoscorpiones lists 49 species (of which one is represented by two subspecies) for Germany (Muster & Blick, 2016). *Lamprochernes savignyi* Simon is a new record for the country, only recently the species has been recorded from Central Europe (Christophoryová et al., 2021). The identity of the specimen from a fallow vineyard in Lorch in Hesse was revealed by the use of the BOLD identification engine (https://www.boldsystems.org/index.php/IDS_OpenIdEngine) which resulted in >99% similarity with public *L. savignyi* sequences. Morphological re‐examination confirmed the determination. For 21 species (58% of the analyzed species), we provide the first publicly available barcodes, including the rare *Anthrenochernes stellae*, a species protected by the FFH Habitats Directive.

**TABLE 1:** List of studied pseudoscorpion species and distance analysis. Except for number of publicly available sequences (all BOLD data), the data refer to the BOLD data set “DS‐GBPSS GBOL‐Pseudoscorpiones Germany”

	Family	Species	*n*	BOLD	BIN	ASAP	MID	DNN	NNS
Chthonioidea	Chthoniidae	*Chthonius alpicola*	1	0	1	1	na	17.07	*E. tetrachelatus*
	Chthoniidae	*Chthonius ischnocheles*	2	8	1	1	na	15.05	*E. boldorii*
	Chthoniidae	*Chthonius tenuis*	1	0	1	1	na	na	
	Chthoniidae	*Ephippiochthonius boldorii*	11	0	1	1	0.56	14.66	*E. fuscimanus*
	Chthoniidae	*Ephippiochthonius fuscimanus*	2	0	1	1	na	14.66	*E. boldorii*
	Chthoniidae	*Ephippiochthonius tetrachelatus*	15	27	2	2	17.33	16.16	*C. ischnocheles*
Neobisioidea	Neobisiidae	*Microbisium brevifemoratum*	2	0	1	1	0.15	12.06	*M. suecicum*
	Neobisiidae	*Microbisium suecicum*	1	0	1	1	na	11.91	*N. carcinoides*
	Neobisiidae	*Neobisium carcinoides*	154	1	23	12	16.79	9.31	*N. hermanni*
	Neobisiidae	*Neobisium erythrodactylum*	7	0	1	1	0.61	12.37	*N. carcinoides*
	Neobisiidae	*Neobisium fuscimanum*	16	0	1	1	1.37	13.89	*N. carcinoides*
	Neobisiidae	*Neobisium hermanni*	4	0	2	1	3.05	9.31	*N. carcinoides*
	Neobisiidae	*Neobisium maritimum*	1	0	1	1	na	16.64	*N. carcinoides*
	Neobisiidae	*Neobisium simile*	10	3	2	1	2.6	17.75	*N. hermanni*
	Neobisiidae	*Neobisium simoni*	3	0	1	1	1.37	13.74	*N. carcinoides*
	Neobisiidae	*Neobisium sylvaticum*	31	0	2	2	9.77	14.2	*N. carcinoides*
	Neobisiidae	*Roncus lubricus*	2	0	1	1	0.48	12.37	*N. carcinoides*
Garypinoidea	Larcidae	*Larca lata*	1	1	1	1	na	24.89	*N. simoni*
Cheiridioidea	Cheiridiidae	*Apocheiridium ferum*	1	0	1	1	na	28.7	*C. cimicoides*
	Cheiridiidae	*Cheiridium museorum*	2	1	1	1	1.07	23.51	*L. chyzeri*
Cheliferoidea	Chernetidae	*Allochernes peregrinus*	15	0	1	1	0.92	18.55	*P. scorpioides*
	Chernetidae	*Allochernes powelli*	4	0	1	1	0.46	17.25	*A. wideri*
	Chernetidae	*Allochernes wideri*	13	0	5	1	4.73	17.25	*A. powelli*
	Chernetidae	*Anthrenochernes stellae*	2	0	1	1	1.53	20.03	*P. dubius*
	Chernetidae	*Chernes cimicoides*	47	2	1	1	1.9	13.46	*C. hahnii*
	Chernetidae	*Chernes hahnii*	9	34	1	1	1.23	13.46	*C. cimicoides*
	Chernetidae	*Chernes nigrimanus*	6	1	1	1	0.36	13.73	*C. hahnii*
	Chernetidae	*Dendrochernes cyrneus*	14	3	4	1	3.82	17.56	*C. hahnii*
	Chernetidae	*Dinocheirus panzeri*	14	2	2	2	12.82	15.57	*C. nigrimanus*
	Chernetidae	*Lamprochernes chyzeri*	9	0	1	1	0.92	16.18	*L. savignyi*
	Chernetidae	*Lamprochernes nodosus*	3	1	1	1	0.35	16.42	*L. chyzeri*
	Chernetidae	*Lamprochernes savignyi*	1	6	1	1	na	16.18	*L. chyzeri*
	Chernetidae	*Pselaphochernes dubius*	9	4	2	1	3.67	17.89	*L. chyzeri*
	Chernetidae	*Pselaphochernes scorpioides*	37	10	2	1	5.57	18.55	*A. peregrinus*
	Cheliferidae	*Chelifer cancroides*	6	3	2	1	3.51	18.93	*D. latreillei*
	Cheliferidae	*Dactylochelifer latreillei*	1	0	1	1	na	18.93	*C. cancroides*

Note

*n*, number of specimens with barcode; BOLD, publicly available sequences on BOLD as of 25th January 2021; BIN, number of BINs per species; ASAP, number of species as proposed by ASAP analysis; MID, maximum intraspecific p‐distance; DNN, minimum interspecific p‐distance to the nearest neighbor species; NNS, nearest neighbor species.

**FIGURE 2:**
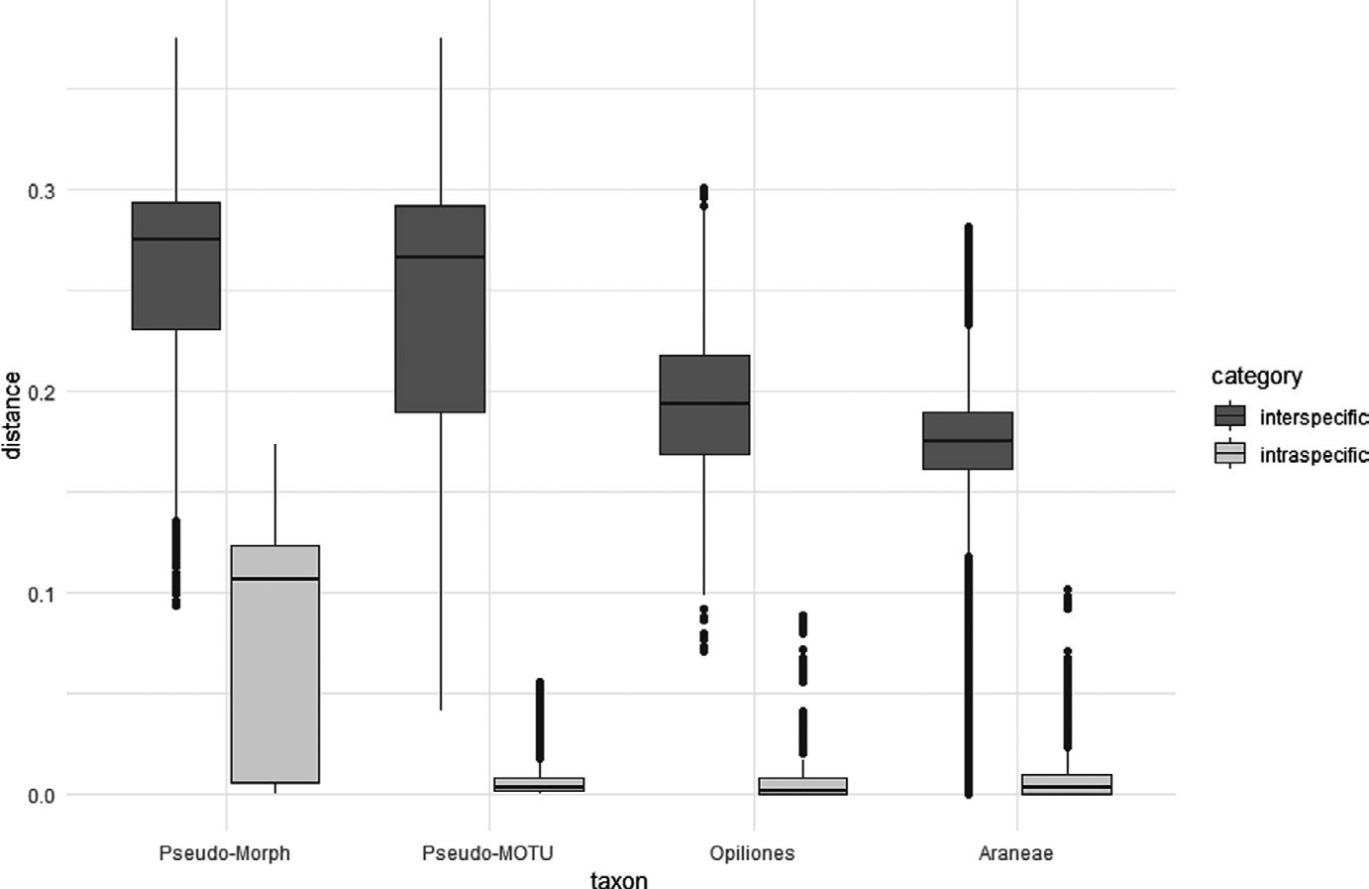
Boxplots showing inter‐ and intraspecific p‐distances in Pseudoscorpiones as compared to Opiliones and Araneae. Pseudo‐Morph—species delineation according to current taxonomy, Pseudo‐MOTU—species delineation according to ASAP. Boxes indicate interquartile range (IQR, between upper [Q3] and lower [Q1] quartiles). Black bars designate medians, whiskers indicate values within 1.5 × IQR beneath Q1 or 1.5 × above Q3. Circles depict outliers (above or below 1.5 × IQR)

**FIGURE 3:**
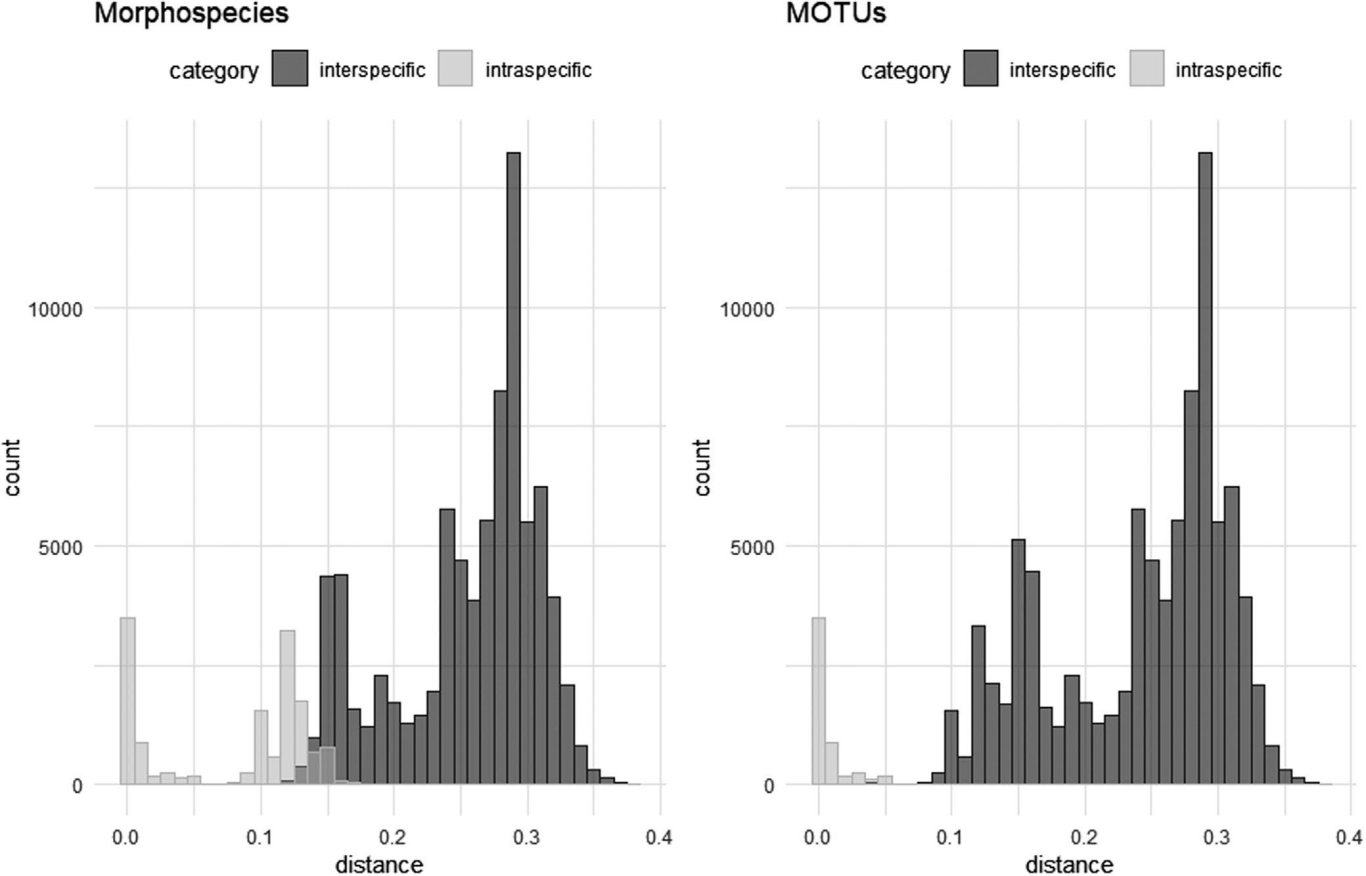
Histograms of intra‐ and interspecific p‐distances in pseudoscorpions as delineated by current taxonomy (morphospecies, left) and ASAP partitioning (MOTUs, right), illustrating the effect of taxonomic misconceptions on the presence of a barcode gap

The median number of generated barcodes per species was four (Table 1). Eight species were covered by single specimens only. For the notoriously puzzling *Neobisium carcinoides* complex, we obtained 154 sequences. The intraspecific p‐distance in pseudoscorpions ranges from 0% to as much as 17.3% when current taxonomy is taken as the basis (mean 8%, median 10.6%; Figure 2). To assess the potential impact of inaccurate taxonomy on our data, we performed a second distance analysis, using the MOTUs as delineated by ASAP as species entities. This resulted in significantly lower intraspecific distances (0%–5.5%, mean 0.75%, median 0.3%), demonstrating the great effect of the underlying taxonomic concept on the manifestation of a barcode gap (Figures 2 and 3).

**FIGURE 4:**
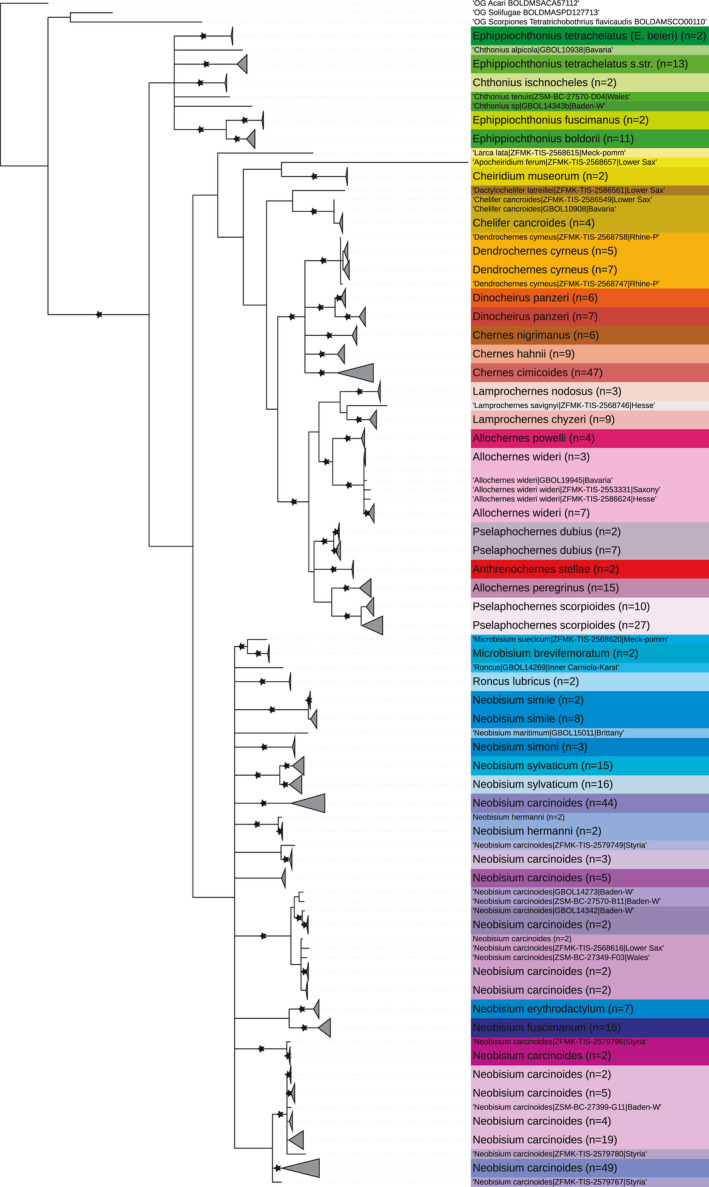
Maximum‐likelihood tree of 459 pseudoscorpion COI sequences from the released dataset and three out‐groups. Branches are collapsed at the level of BINs, and the width of the triangles is proportional to the number of specimens in a branch. Asterisks indicate nodes with bootstrap support ≥95% (10,000 replicates). ASAP partitions are shown in different colors. The tree was annotated with iTOL v4 (Letunic & Bork, 2019)

Generally, we found high congruence between the pattern of COI variation and morphology‐based identifications: 37 species (95%) were recovered monophyletic in the ML tree (Figure 4, see Appendix S2 for a fully resolved tree). Only *Ephippiochthonius tetrachelatus* s.l. and *Neobisium carcinoides* s.l. were polyphyletic (see comments on these taxa below). Also, phylogenetic relationships above the species level correspond fairly well with morphology‐based hypotheses, as the majority of traditional genera form monophyletic clusters. Exceptions include (i) *Roncus*, which is paraphyletic with respect to *Neobisium* and (ii) *Allochernes* and *Pselaphochernes*, which appear polyphyletic. Also, the recently elevated genus *Ephippiochthonius* is not monophyletic in our reconstructions, casting doubt on the generic rank of previous subgenera within *Chthonius* (while keeping in mind our phylogenetic reconstruction is based on a single gene).

The sequences of our 36 morphospecies were allocated to 74 BINs. BIN excess is thus 105% ([*n* BINs – *n* species]/*n* species x 100). Nevertheless, two thirds of the pseudoscorpion species (*n* = 25) were BIN concordant; that is, all barcodes of these species belonged to a single BIN. Barcodes from species with BIN divergence (multiple BINs within a species) were allocated to two BINs (*Chelifer cancroides* (Linnneaus)*,* *Ephippioc* *hthonius tetrachelatus* (Preyssler)*, Dinocheirus panzeri* (C.L. Koch)*, Neobisium hermanni* Beier*, Neobisium simile* (L. Koch)*, Neobisium sylvaticum* (C.L. Koch)*, Pselaphochernes dubius* (O.P.‐Cambridge)*, Pselaphochernes scorpioides* (Hermann)), four BINs (*Dendrochernes cyrneus* (L. Koch)) or five BINs (*Allochernes wideri* (C.L. Koch)). A noticeable exception is *Neobisium carcinoides* (Hermann), whose COI sequences were assigned to 23 BINs.

The level of intraspecific COI variation was not generally correlated with ecological guilds. Several epigeic species with limited dispersal capacity show little genetic variation across their range. Quite unexpected was the finding of absolutely identical haplotypes in 13 specimens of *Ephippiochthonius tetrachelatus* s. str. that were collected between southwestern Germany and the Baltic Sea coast. Shallow phylogeographic structure across large areas was also found in several epigeic *Neobisium* species with distinct morphology, for example, *N. fuscimanum* (C.L. Koch) and *N. erythrodactylum* (L. Koch). The phoretic chernetids include species with very low intraspecific variation, for example, *Chernes cimicoides* (Fabricius) (mean intraspecific p‐distance 0.48%, max. 1.9, *n* = 47) and species with distinct phylogeographic lineages, for example, *Allochernes wideri* (mean intraspecific p‐distance 2.42%, max. 4.73, *n* = 13).

**FIGURE 5:**
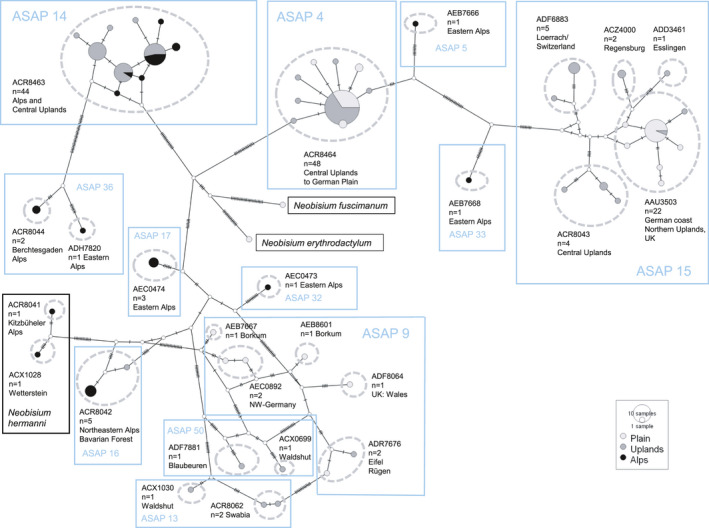
COI haplotype network for *Neobisium carcinoides* s.l. Among 154 sequences of *N. carcinoides* and the closely related *N. hermanni*, 55 different haplotypes were recorded. The most frequent haplotype was found in 38 specimens. Sequences originate from the plain (44), the central uplands (82) and the Alps (28). BINs are shown by dashed ellipses (23 in *N. carcinoides*, 2 in *N. hermanni*). Species delineations as proposed by ASAP are shown in blue rectangles (12 in *N. carciniodes*). The network was constructed with the TCS algorithm using PopART version 1.7 (www.popart.otago.ac.nz)

Species delineation with the best ASAP score (5.50) was achieved at a distance threshold of 5.5% (p‐distance). This partition predicts 51 putative species (MOTUs). Species limits are fully congruent with traditional taxonomy, except for *Ephippiochthonius tetrachelatus, Neobisium sylvaticum,* and *Dinocheirus panzeri*, which are split into two MOTUs each, and *Neobisium carcinoides*, which is divided into 12 MOTUs (Figures 4 and 5). The partition with the second‐best ASAP score (7.50, distance threshold 4%) delimits exactly the same groups, with exception of splitting up two sequences of *Neobisisum caricnoides* from Baden Württemberg in separate partitions (ASAP 13 in Figure 5), summing up to 52 candidate species (Appendix S3). The close similarity among the best ASAP partitions indicates the robustness of the results.

**FIGURE 6:**
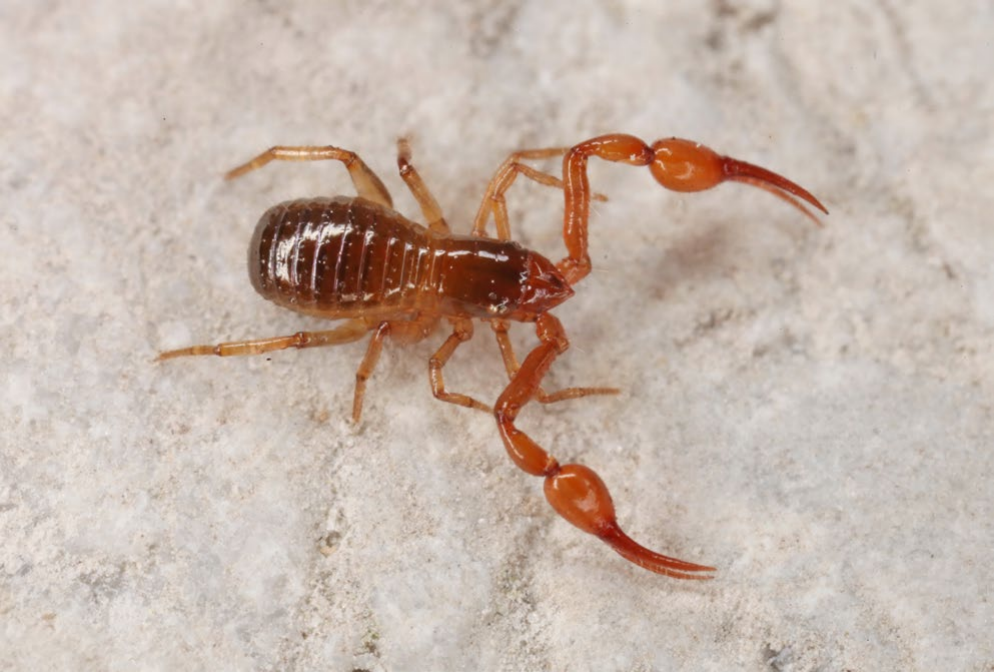
Male of *Neobisium carcinoides* in the Gesäuse National Park. In this area, we recorded seven species‐level lineages within this “polymorphic” species. Photo by Christian Komposch

**FIGURE 7:**
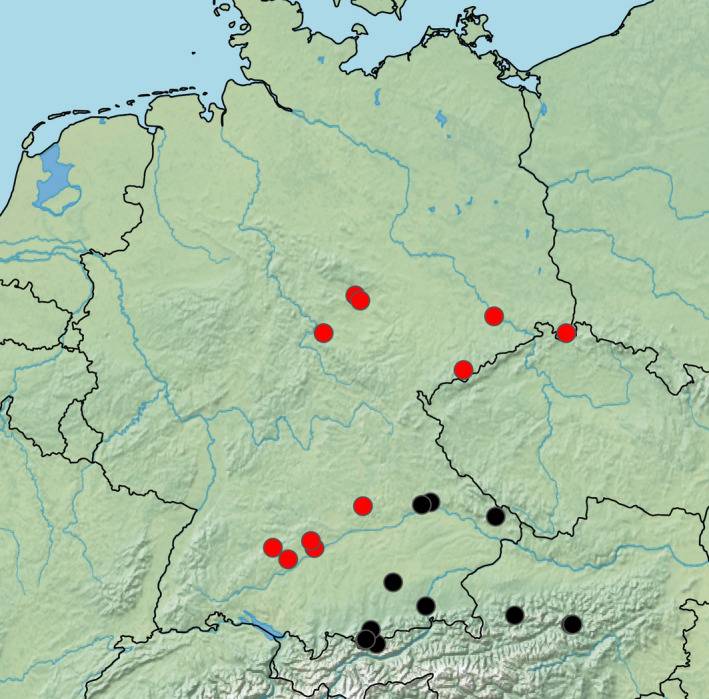
Allopatric distribution of two deeply diverged clades (COI distance 9.3%) of *Neobisium sylvaticum* in Germany (and Austria). Red dots: localities of northwestern lineage; black dots: localities of southeastern lineage. The map was produced with SimpleMappr (www.simplemappr.net)

**FIGURE 8:**
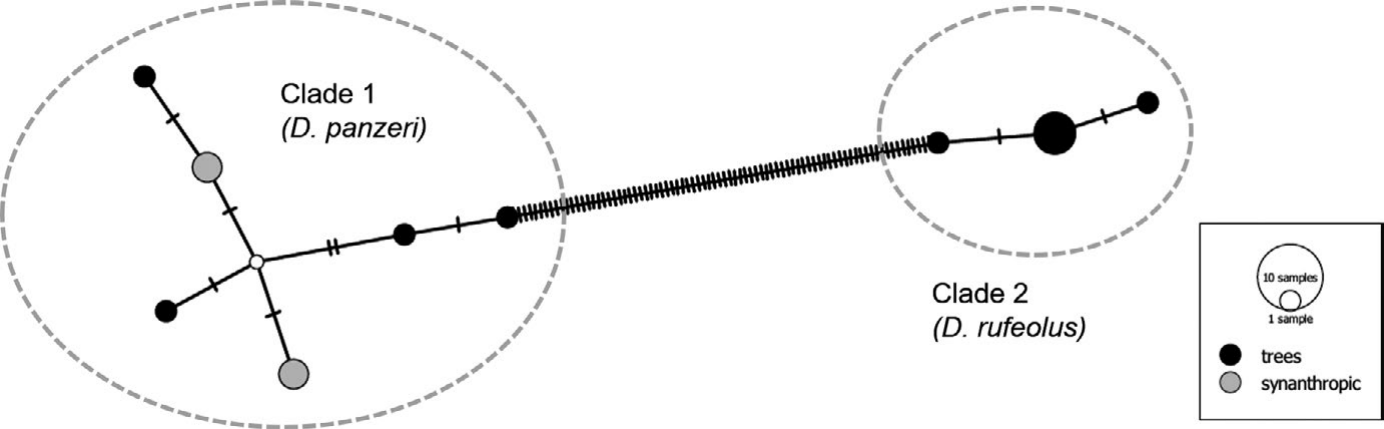
COI haplotype network for two species currently standing as *Dinocheirus panzeri*. The p‐distance between the lineages is 12.5%. Both lineages dwell in the mold of tree hollows, while only *D. panzeri* s. str. occurs in barns, cattle sheds, and compost heaps. The network was constructed with the TCS algorithm using PopART version 1.7 (www.popart.otago.ac.nz)

*Neobisium carcinoides* (Figure 6) had the highest intraspecific diversity among European pseudoscorpions. The 154 COI sequences in the GBOL dataset correspond to 12–23 putative species, depending on the method used for delineation of OTUs (Figure 5). North of the Alps we found three MOTUs to occur up to the North German Plain, and three additional lineages up to the northern limits of the Hercynian mountains. However, the situation is much more complex in the Alps, where our limited sampling revealed numerous unique lineages of unknown range. For example, the analysis of ten *N. carcinoides* sequences from the National Park Gesäuse (Eastern Alps, Austria) suggests allocation to seven species‐level lineages of sympatric occurrence. Another taxon with a deep intraspecific split is *Neobisium sylvaticum*. In this case, the sequences are distributed fairly evenly among only two lineages (Figure 4), with a mean p‐distance of 9.3%, but little variation within the lineages (max. within‐clade distance <1%). The available data suggest an allopatric distribution pattern, with a northwestern lineage bordering a southeastern lineage in Bavaria (Figure 7). Also in the chernetid *Dinocheirus panzeri,* the COI sequences fall into two deeply diverged clades. These are separated from each other by 81 mutations (Figure 8). The mean p‐distance between the clades is 12.5%. Specimens from synanthropic sites were restricted to clade 1, while specimens from the mold of trees were distributed across both clades.

## 4 DISCUSSION

The GBOL dataset is the most comprehensive regional barcode library for Pseudoscorpiones published to date, comprising 459 sequences from 36 species. German fauna coverage of the GBOL dataset is 69% for Pseudoscorpiones, this is, more than in spiders (57%) and similar to harvestmen (71%) (Astrin et al., 2016). A striking feature of the pseudoscorpion dataset is the high discrepancy between Linnean taxa (morphospecies) and BIN assignments, almost entirely attributable to BIN divergence, that is, the detection of multiple BINs within Linnean species. BIN/species ratios larger than 1.0 usually indicate the presence of species that are overlooked by the current taxonomic system (Hebert et al., 2016). In our dataset, the number of BINs is almost double the number of documented species, and similar results were obtained for Canadian pseudoscorpions (Cameron & Buddle, 2019). BIN excess was much lower in other well‐studied groups in GBOL, for example, 5% in beetles (Hendrich et al., 2015), 13% in grasshoppers (Hawlitschek et al., 2017), and 34% in water striders (Havemann et al., 2018). Only in Diptera, a hyperdiverse but notoriously understudied taxon, a similar value of 112% was recovered (Morinière et al., 2019). Of particular interest is the extraordinarily high BIN diversity within the Moss neobisid *Neobisium carcinoides* (23 BINs within one Linnean species). Similar examples have rarely been reported from terrestrial arthropods. Among >1,000 analyzed species of Canadian spiders, three were assigned to more than 10 BINs, with the dwarf spider *Grammonota angusta* Dondale achieving the highest BIN count of 22 (Blagoev et al., 2016). Most BINs of *G. angusta* show narrow geographic distributions in eastern Canada, resembling the observed pattern for *N. carcinoides* in the Alps. Thus, in both cases the observed variation could result from diversification in allopatric glacial area isolates. Hyper‐cryptic diversity is more frequently found within morphospecies of freshwater amphipods. Recently, Wattier et al. (2020) identified 146 BINs within *Gammarus fossarum* Koch, 1836. They consider the taxonomic impediment, morphological stasis, or parallel evolution as possible explanations for the exceptional high level of hidden diversity in this widespread amphipod.

High intraspecific sequence divergence in pseudoscorpions may result from undocumented species diversity, but also from anomalies in the COI evolution of these arachnids. There is growing evidence for accelerated rates of molecular evolution in the mitochondrial and nuclear genome of pseudoscorpions (Arabi et al., 2012). In fact, pseudoscorpions showed the largest order distance (divergence to the out‐group *Limulus polyphemus*) and the largest BIN distance (mean divergence among BINs within families) among all arachnids (Young & Hebert, 2015). Pseudoscorpiones have a higher occurrence of fixed mutations of all types, including base substitutions, insertions/deletions, and genomic rearrangements, and this may distort the pattern of intra‐ and interspecific variation, that is, the existence of a barcode gap.

Our empirical data, however, do not signal principal constraints for identification and delineation of pseudoscorpion species through DNA barcoding, as we did not find any incidences of BIN sharing (where BINs would comprise sequences from more than one Linnean species). True incidences of BIN sharing (excluding operational factors such as inaccurate taxonomy, misidentification, NUMT amplification) are most frequently caused by incomplete lineate sorting and hybridization among phylogenetically young species (Hawlitschek et al., 2017). These biological processes pose challenge to any species delineation method, particularly if based on single‐locus data (Puillandre et al., 2021). However, we detected no signs for confounding effects attributable to those processes in European pseudoscorpions.

Taking our initial assignment to Linnean species into account (based on the currently accepted taxonomy), then the intraspecific diversity was indeed higher in pseudoscorpions (mean 8%, median 10.6%) than in spiders (mean 0.7%, median 0.3%) and harvestmen (mean 1.3%, median 0.2%) from the GBOL project (Astrin et al., 2016). Also, the interspecific distances (9.3%–37.5%, mean 25.6%, median 27.5%) were generally higher than in spiders (mean 17.4%, median 17.5%) and harvestmen (mean 19.4%, median 19.3%). Intra‐ and interspecific distances overlapped, thus spoiling a barcode gap (Figures 2, 3). However, this result may be strongly affected by inaccurate reference taxonomy, that is, the presence of either overlooked cryptic or oversplit species. Mutanen et al. (2016) have demonstrated the significant impact of taxonomic misinterpretation on estimates of species monophyly in COI barcode gene trees, and our pseudoscorpion data provide an additional example. When taxonomy is based on ASAP‐MOTUs, the intraspecific distances in pseudoscorpions (0%–5.5%, mean 0.75%, median 0.3%) fall into a similar range as in other arachnids, while interspecific distances remain higher (mean 24.3%, median 26.6%). The median distance to the closest species (median of all comparisons between species pairs) was 16.4% for the traditional morphospecies and 14.7% for the ASAP‐MOTUs, which is still higher than for spiders (9.2%) and harvestmen (13.8%). Therefore, this analysis shows not only the presence of a barcode gap, but that such a gap between intra‐ and interspecific distances may even be larger in pseudoscorpions than in other arachnids and insects. The ASAP estimate of 51 species in our dataset means a 42% increase in species number compared with the current taxonomy. We highlight that ASAP estimates the distance threshold that is used for partition of the samples into putative species from the data. In our dataset, the best ASAP score was achieved at a distance threshold of 5.5%, showing that the accelerated substitution rate in pseudoscorpions has been taken into account. On the other hand, we explicitly warn against the use of BIN clusters as a proxy of species diversity in pseudoscorpions, as the underlying RESL algorithm is based on a predefined distance threshold of 2.2%.

We argue that the species delineation as proposed by ASAP accurately reflects true species boundaries in European pseudoscorpions. In all cases of incongruence between traditional taxonomy and ASAP‐MOTUs, there is independent morphological and/or karyological evidence for cryptic speciation, and available Linnean names wait to be released from synonymy. For example, the two distinct haplotypes of *Ephippiochthonius tetrachelatus* s.l. from Baden‐Wuerttemberg (ZSM‐BC‐27399‐D05, GBOL10925), which cluster in a distant BIN, carry two lateral microsetae at the posterior margin of the carapace. They thus correspond morphologically to *Ephippiochthonius beieri* (Lazzeroni). Gardini (2013) “temporarily” considered *E. beieri* to be a junior synonym of *E. tetrachelatus* but noticed “*tetrachelatus* probably hides a species complex that current morphological taxonomy is unable to detect.” With respect to the Terrible‐clawed chernes, there has been a long debate whether *D. panzeri* and *D. rufeolus* (Simon) were separate species, ecological races, or just the two poles in the range of continuous intraspecific variation (Ressl, 1983). Drogla and Lippold (2004) demonstrate the ecological divergence: *Dinocheirus panzeri* sensu lato was as widespread in mold of tree cavities as it was in synanthropic habitats (barns, dung, and compost heaps). Beier (1963) listed both species in different genera, sub *Toxochernes panzeri* and *Chernes rufeolus*, but he later changed his opinion and considered them synonyms (Beier in litt., 1973; see Ressl, 1983:192), as first proposed by Ellingsen (1907) and later confirmed by Mahnert (1978). These authors justified the synonymization by the presence of transitional morphotypes in the male and indistinguishability of the females. However, mitochondrial variation is anything but continuous and clearly calls for acceptance of two species. Among the examined males, only those of clade 2 show the longitudinal depression at the medial side of the palpal chela, a characteristic trait of *D*. *rufeolus* (Beier, 1963). Therefore, we are confident that the specimens from clade 2 should be attributed to *Dinocheirus rufeolus*. However, formal reinstatement of this species calls for a thorough taxonomic treatment, including consideration of type material, which cannot be achieved here. The same holds true for the putative sibling species within *Neobisium sylvaticum*. Harvey (2013) lists several available names that are currently considered synonyms of *N. sylvaticum* C.L. Koch, 1839 (type locality Regensburg and Frauenholz), that is, *N*. *dumicola* C.L. Koch, 1835 (locus typicus Regensburg, Bavaria), *N*. *walckenaerii* Théis, 1832 (type locality St. Gobain, France), *N*. *elimatum* C.L. Koch, 1839 (type locality Bavaria), and *N*. *dubium* C.L. Koch, 1843 (type locality Nürnberg, Bavaria).

*Neobisium carcinoides* is a unique case that can only be settled in an in‐depth study following an integrative taxonomy approach which includes molecular, karyological, and morphometric data. The World Catalogue of pseudoscorpions (Harvey, 2013) lists eight taxa in synonymy of *N. carcinoides*. Up to the 1950s, three common and widespread species of this complex have been distinguished in Central Europe; these are *N. carcinoides* (Hermann), *N. muscorum* (Leach), and *N. germanicum* Beier. Guided by the observation of large and superficially haphazard variation, Beier (1963) proposed synonymy of these species, and this conclusion was approved by Mahnert (1988), although with certain doubt: “Et cette hypothèse me semble plus raisonnable que de décrire une dizaine ou une vingtaine d'espèces locales, difficilement différenciables sans connaître leur provenance géographique” [And this hypothesis seems more reasonable to me than describing ten or twenty local species, difficult to differentiate without knowing their geographic origin]. Reasonable doubts as to the accuracy of the polymorphic species hypothesis have already been raised by phenological (Meyer et al., 1985) and preliminary karyological studies (Šťáhlavský et al., 2003). Our genetic data clearly support the competing hypothesis of 10–20 cryptic species within a morphologically conserved complex. The taxonomic revision of this species aggregate constitutes a major challenge, given the high number of available names and the magnitude of sympatric occurrences. While DNA barcoding is effective in establishing interim taxonomic systems (Morinière et al., 2019), the inclusion of genomic data may be required to revise such problematic species complexes.

Pseudoscorpion research may particularly benefit from DNA barcoding for a variety of reasons. First, morphological evolution in pseudoscorpions is slow and conservative. The retention of ancestral morphology—despite rapid genetic differentiation—often makes it difficult to distinguish closely related species on the basis of morphological features alone (Šťáhlavský et al., 2009). In addition, certain morphological characters once in use to diagnose pseudoscorpion species have been proven to be unreliable in species delineation (Ohira et al., 2018). Even specialists of the group may face difficulties in identification of certain specimens and thus make use of the identification tool on BOLD. Second, recruitment and training of specialists for the group is challenging, among other factors caused by the cryptic morphology. Availability of a comprehensive barcode library will make the taxon more attractive to interested candidates and thus contribute to continuity and advancement of professional knowledge. Third, pseudoscorpions are difficult to collect. They are small animals, often camouflaged in appearance and living in inaccessible microhabitats, such as rock crevices, the mesovoid shallow substratum (MSS), burrows of mammals, bird nests, or tree hollows in canopies (Bedoya‐Roqueme & Tizo‐Pedroso, 2021). Field‐based research on pseudoscorpions may therefore profit from environmental DNA metabarcoding, that is, the detection of species in genetic material from environmental samples (Ruppert et al., 2019). This appears particularly relevant with respect to the monitoring of *Anthrenochernes stellae*, a pseudoscorpion species protected by the European Flora‐Fauna‐Habitat directive (Council Directive 92/43/ EEC on the Conservation of natural habitats and of wild fauna and flora, 1992). Article 11 of the Habitats Directive obliges EU member states to monitor the conservation status of natural habitats (Annex I) and species of European interest (Annex II, IV, and V). Surveys using conventional methods to record this Annex II species regularly fail, because *Anthrenochernes stellae* is extremely rare and dwells in tree hollows that are hard to access (Holmen & Scharff, 2008). DNA extraction from mold/litter samples from tree cavities offers a promising perspective for surveillance of this species. Forth, the BIN and ASAP partitions provide an interim taxonomic system on which thorough taxonomic revisions of morphologically uniform species groups can be based. Re‐examination of distinct DNA lineages within morphospecies often resulted in the detection of diagnostic but previously overlooked morphological characters, thus releasing the involved species from morphological crypsis (Lin et al., 2018; Muster & Michalik, 2020; Stüben & Astrin, 2010; Wachter et al., 2015). It should be noted that some problematic taxa among the European pseudoscorpions are strongly underrepresented in the GBOL sampling. This is particularly true for some species in *Chthonius* s. str.; for example, *Chthonius tenuis* L. Koch. Kotrbová et al. (2016) detected several cytotypes among *C. tenuis* populations in the Alps. And even the leading expert for this group, Giulio Gardini, failed to allocate specimens from Germany unequivocally to either *C. tenuis* or *C*. *submontanus* Beier: “I have redescribed both *C. tenuis* and *C. submontanus* in 2009, but I see now that it is not enough to understand these species” (Gardini in litt., 2017). There is still a lot of hidden diversity to be discovered, even in comparatively well‐explored regions, and DNA barcoding may pave that way.

## CONFLICT OF INTEREST

The authors declare they have no conflict of interests.

## AUTHOR CONTRIBUTION

**Christoph Muster:** Conceptualization (supporting); Formal analysis (lead); Investigation (supporting); Validation (supporting); Visualization (lead); Writing‐original draft (lead); Writing‐review & editing (lead). **Jörg Spelda:** Conceptualization (equal); Data curation (equal); Funding acquisition (equal); Investigation (supporting); Project administration (equal); Resources (equal); Writing‐review & editing (supporting). **Björn Rulik:** Conceptualization (supporting); Data curation (equal); Investigation (supporting); Project administration (supporting); Supervision (supporting). **Jana Thormann:** Data curation (equal); Investigation (equal); Project administration (supporting). **Laura von der Mark:** Data curation (equal); Investigation (equal); Project administration (supporting). **Jonas J. Astrin:** Conceptualization (equal); Data curation (supporting); Formal analysis (equal); Funding acquisition (lead); Investigation (supporting); Methodology (equal); Project administration (lead); Resources (lead); Software (equal); Supervision (supporting); Validation (supporting); Visualization (supporting); Writing‐review & editing (equal).

### OPEN RESEARCH BADGES

This article has earned an Open Data Badge for making publicly available the digitally‐shareable data necessary to reproduce the reported results. The data is available at https://doi.org/10.5883/DS‐GBPSS.

## Supporting information

Appendix S1‐S4

## Data Availability

Sequence data are available in the public data set “DS‐GBPSS GBOL‐Pseudoscorpiones Germany” (http://doi.org/10.5883/DS‐GBPSS) on BOLD and on GenBank (acc‐nos MN621854‐MN621856, MW995987‐MW996442). The final DNA sequence alignment and specimen locality data were uploaded as online supplements.
