# Supplementary material for: The dark side of pseudoscorpion diversity: The German Barcode of Life campaign reveals high levels of undocumented diversity in European false scorpions
Source: Ecol Evol. 2021 Sep 8;11(20):13815–29. doi: 10.1002/ece3.8088 (PMC8525104; doi:10.1002/ece3.8088)

Supplemental information for:
The dark side of pseudoscorpion diversity:
the German Barcode of Life campaign reveals high levels
of undocumented diversity in European false scorpions

Christoph Muster, Jörg Spelda, Björn Rulik, Jana Thormann,
Laura von der Mark, Jonas J. Astrin

Appendix S2: Maximum likelihood tree of 459 sequences
from the BOLD dataset "DS-GBPSS GBOL-Pseudoscorpiones Germany"

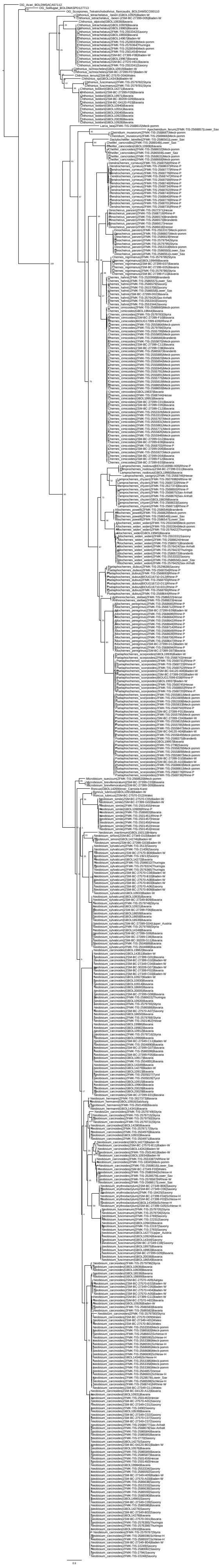

Supplement: Supplementary file 1 — Appendix S1‐S4 [file ECE3-11-13815-s001.zip › ece38088-sup-0002-AppendixS2.pdf]
