# Supplementary material for: The dark side of pseudoscorpion diversity: The German Barcode of Life campaign reveals high levels of undocumented diversity in European false scorpions
Source: Ecol Evol. 2021 Sep 8;11(20):13815–29. doi: 10.1002/ece3.8088 (PMC8525104; doi:10.1002/ece3.8088)
Supplement: Supplementary file 1 — Appendix S1‐S4 [file ECE3-11-13815-s001.zip › ece38088-sup-0003-AppendixS3.pdf]

Supplemental information for:

The dark side of pseudoscorpion diversity: the German Barcode of Life campaign reveals high levels of undocumented diversity in European false scorpions

Christoph Muster, Jörg Spelda, Björn Rulik, Jana Thormann, Laura von der Mark, Jonas J. Astrin

Appendix S3: Results from ASAP partition analysis

Input: 440 sequences > 500 bp

Substitution model: p-distance

Table S3-1: Scores at the 10 best partitions found by ASAP

Nb of species	asap-score	P-val (rank)		W (rank)	Treshold dist.
51	5.50		1.30e-02 (8)	2.41e-04 (3)	0.054610
* 52	7.50		3.00e-05 (4)	7.11e-05 (11)	0.040458
46	8.00		1.00e-05 (3)	5.80e-05 (13)	0.084781
49	11.00		4.13e-01 (20)	4.20e-04 (2)	0.071756
* 53	11.50		1.06e-01 (13)	7.15e-05 (10)	0.038101
* 70	11.50		1.60e-01 (14)	9.26e-05 (9)	0.021325
* 73	13.50		4.17e-01 (21)	1.25e-04 (6)	0.016794
* 54	14.50		2.57e-01 (17)	6.79e-05 (12)	0.035048
* 61	15.00		3.90e-02 (10)	4.00e-05 (20)	0.028681
* 60	16.50		4.23e-02 (11)	3.71e-05 (22)	0.030207

Nb – number of species partitions
asap-score = (rank [P-val] + rank [W]) / 2
P-val – probability of panmixia
W – relative gap width

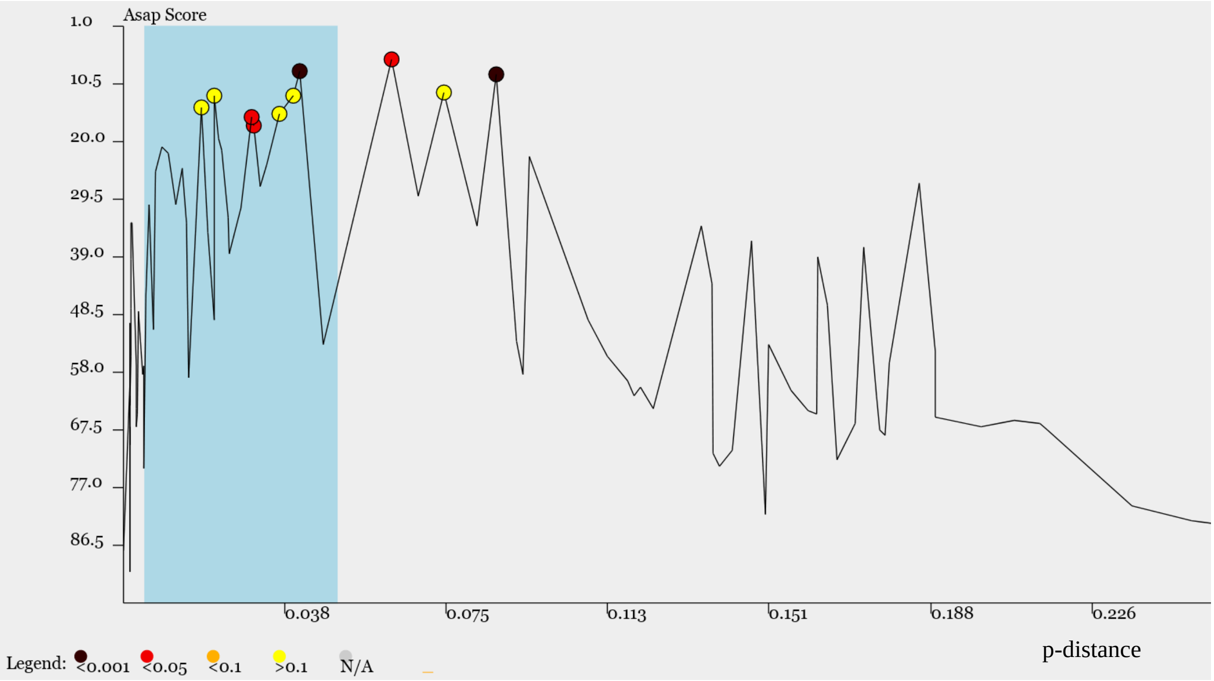

Fig. S3-1 Curve of ASAP scores at p-distances from 0.1 – 25%

Reference

Puillandre, N., Brouillet, S., & Achaz, G. (2021). ASAP: assemble species by automatic partitioning. *Molecular Ecology Resources, 21,* 609–620. https://doi.org/10.1111/1755-0998.13281

Fig S3-2: Dendrogram with ASAP partitions at the 10 best scores

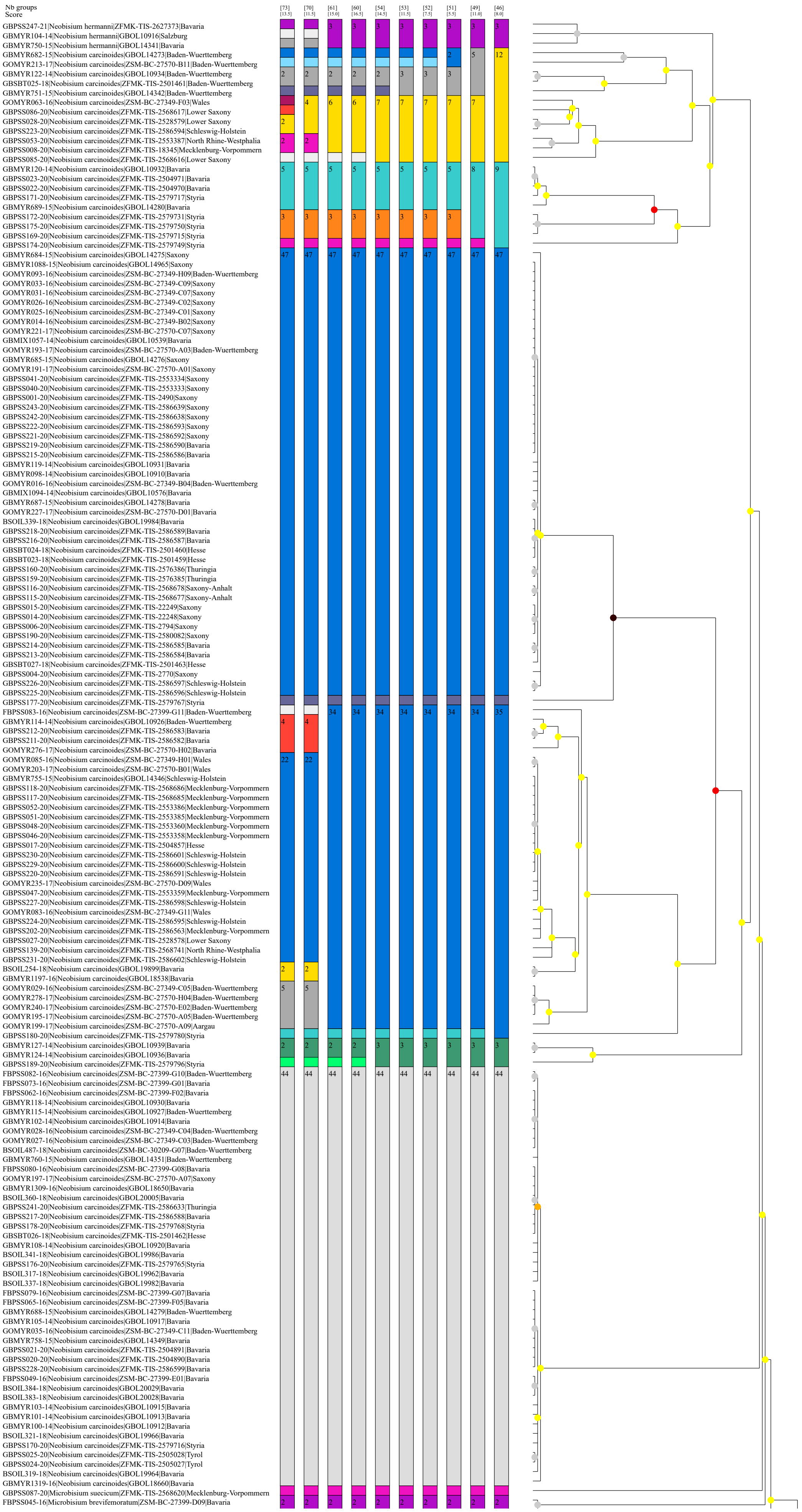

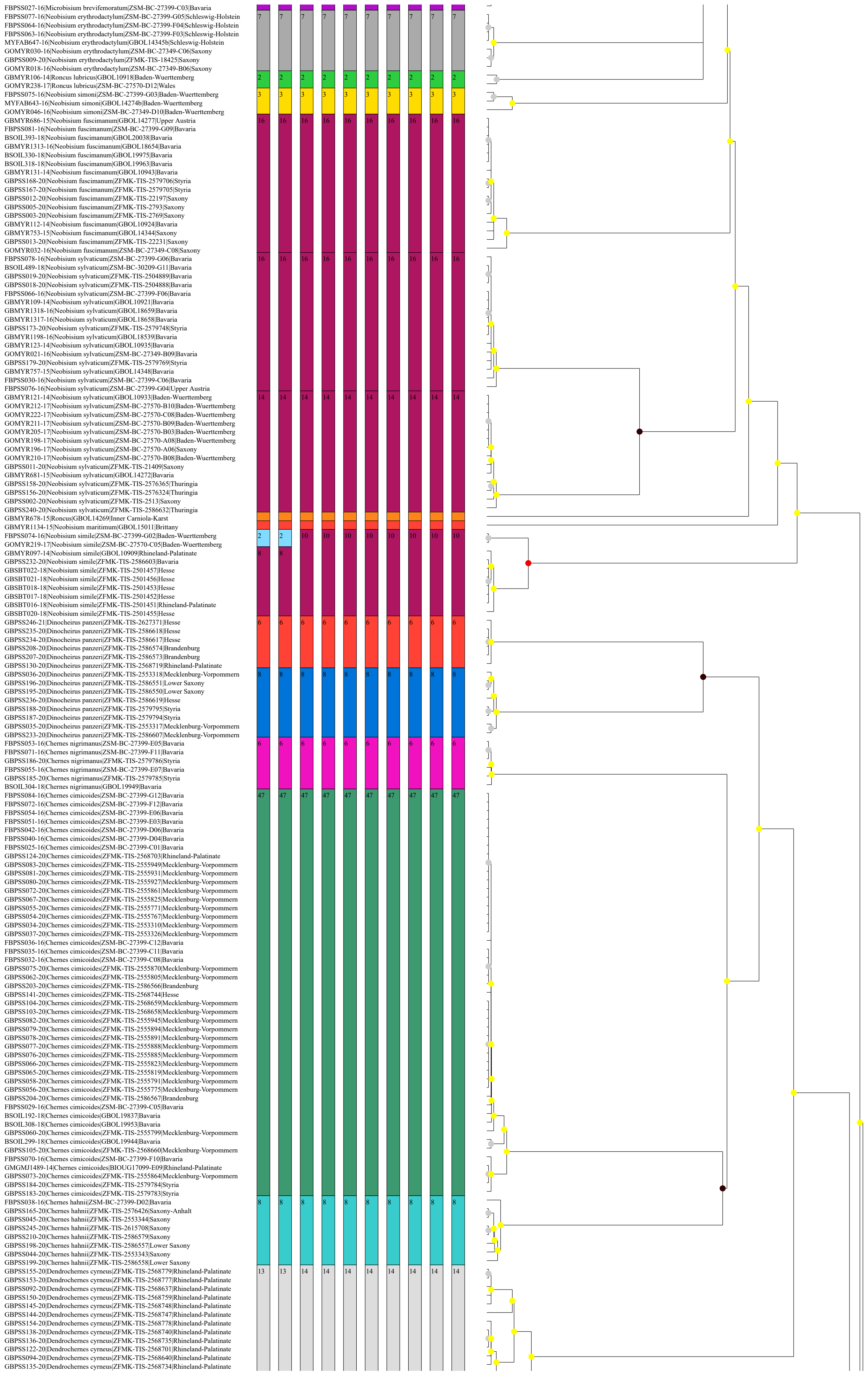
FBPSS027-16|Microbisium brevifemoratum|ZSM-BC-27399-C03|Bavaria
FBPSS077-16|Neobisium erythrodactylum|ZSM-BC-27399-G05|Schleswig-Holstein
FBPSS064-16|Neobisium erythrodactylum|ZSM-BC-27399-F04|Schleswig-Holstein
FBPSS063-16|Neobisium erythrodactylum|ZSM-BC-27399-F03|Schleswig-Holstein
MYFAB647-16|Neobisium erythrodactylum|GBOL14345b|Schleswig-Holstein
GOMYR030-16|Neobisium erythrodactylum|ZSM-BC-27349-C06|Saxony
GBPSS009-20|Neobisium erythrodactylum|ZFMK-TIS-18425|Saxony
GOMYR018-16|Neobisium erythrodactylum|ZSM-BC-27349-B06|Saxony
GBMYR106-14|Roncus lubricus|GBOL10918|Baden-Wuerttemberg
GOMYR238-17|Roncus lubricus|ZSM-BC-27570-D12|Wales
FBPSS075-16|Neobisium simoni|ZSM-BC-27399-G03|Baden-Wuerttemberg
MYFAB643-16|Neobisium simoni|GBOL14274b|Baden-Wuerttemberg
GOMYR046-16|Neobisium simoni|ZSM-BC-27349-D10|Baden-Wuerttemberg
GBMYR686-15|Neobisium fuscimanum|GBOL14277|Upper Austria
FBPSS081-16|Neobisium fuscimanum|ZSM-BC-27399-G09|Bavaria
BSOIL393-18|Neobisium fuscimanum|GBOL20038|Bavaria
GBMYR1313-16|Neobisium fuscimanum|GBOL18654|Bavaria
BSOIL330-18|Neobisium fuscimanum|GBOL19975|Bavaria
BSOIL318-18|Neobisium fuscimanum|GBOL19963|Bavaria
GBMYR131-14|Neobisium fuscimanum|GBOL10943|Bavaria
GBPSS168-20|Neobisium fuscimanum|ZFMK-TIS-2579706|Styria
GBPSS167-20|Neobisium fuscimanum|ZFMK-TIS-2579705|Styria
GBPSS012-20|Neobisium fuscimanum|ZFMK-TIS-22197|Saxony
GBPSS005-20|Neobisium fuscimanum|ZFMK-TIS-2793|Saxony
GBPSS003-20|Neobisium fuscimanum|ZFMK-TIS-2769|Saxony
GBMYR112-14|Neobisium fuscimanum|GBOL10924|Bavaria
GBMYR753-15|Neobisium fuscimanum|GBOL14344|Saxony
GBPSS013-20|Neobisium fuscimanum|ZFMK-TIS-22231|Saxony
GOMYR032-16|Neobisium fuscimanum|ZSM-BC-27349-C08|Saxony
FBPSS078-16|Neobisium sylvaticum|ZSM-BC-27399-G06|Bavaria
BSOIL489-18|Neobisium sylvaticum|ZSM-BC-30209-G11|Bavaria
GBPSS019-20|Neobisium sylvaticum|ZFMK-TIS-2504889|Bavaria
GBPSS018-20|Neobisium sylvaticum|ZFMK-TIS-2504888|Bavaria
FBPSS066-16|Neobisium sylvaticum|ZSM-BC-27399-F06|Bavaria
GBMYR109-14|Neobisium sylvaticum|GBOL10921|Bavaria
GBMYR1318-16|Neobisium sylvaticum|GBOL18659|Bavaria
GBMYR1317-16|Neobisium sylvaticum|GBOL18658|Bavaria
GBPSS173-20|Neobisium sylvaticum|ZFMK-TIS-2579748|Styria
GBMYR1198-16|Neobisium sylvaticum|GBOL18539|Bavaria
GBMYR123-14|Neobisium sylvaticum|GBOL10935|Bavaria
GOMYR021-16|Neobisium sylvaticum|ZSM-BC-27349-B09|Bavaria
GBPSS179-20|Neobisium sylvaticum|ZFMK-TIS-2579769|Styria
GBMYR757-15|Neobisium sylvaticum|GBOL14348|Bavaria
FBPSS030-16|Neobisium sylvaticum|ZSM-BC-27399-C06|Bavaria
FBPSS076-16|Neobisium sylvaticum|ZSM-BC-27399-G04|Upper Austria
GBMYR121-14|Neobisium sylvaticum|GBOL10933|Baden-Wuerttemberg
GOMYR212-17|Neobisium sylvaticum|ZSM-BC-27570-B10|Baden-Wuerttemberg
GOMYR222-17|Neobisium sylvaticum|ZSM-BC-27570-C08|Baden-Wuerttemberg
GOMYR211-17|Neobisium sylvaticum|ZSM-BC-27570-B09|Baden-Wuerttemberg
GOMYR205-17|Neobisium sylvaticum|ZSM-BC-27570-B03|Baden-Wuerttemberg
GOMYR198-17|Neobisium sylvaticum|ZSM-BC-27570-A08|Baden-Wuerttemberg
GOMYR196-17|Neobisium sylvaticum|ZSM-BC-27570-A06|Saxony
GOMYR210-17|Neobisium sylvaticum|ZSM-BC-27570-B08|Baden-Wuerttemberg
GBPSS011-20|Neobisium sylvaticum|ZFMK-TIS-21409|Saxony
GBMYR681-15|Neobisium sylvaticum|GBOL14272|Bavaria
GBPSS158-20|Neobisium sylvaticum|ZFMK-TIS-2576365|Thuringia
GBPSS156-20|Neobisium sylvaticum|ZFMK-TIS-2576324|Thuringia
GBPSS002-20|Neobisium sylvaticum|ZFMK-TIS-2513|Saxony
GBPSS240-20|Neobisium sylvaticum|ZFMK-TIS-2586632|Thuringia
GBMYR678-15|Roncus|GBOL14269|Inner Carniola-Karst
GBMYR1134-15|Neobisium maritimum|GBOL15011|Brittany
FBPSS074-16|Neobisium simile|ZSM-BC-27399-G02|Baden-Wuerttemberg
GOMYR219-17|Neobisium simile|ZSM-BC-27570-C05|Baden-Wuerttemberg
GBMYR097-14|Neobisium simile|GBOL10909|Rhineland-Palatinate
GBPSS232-20|Neobisium simile|ZFMK-TIS-2586603|Bavaria
GBSBT022-18|Neobisium simile|ZFMK-TIS-2501457|Hesse
GBSBT021-18|Neobisium simile|ZFMK-TIS-2501456|Hesse
GBSBT018-18|Neobisium simile|ZFMK-TIS-2501453|Hesse
GBSBT017-18|Neobisium simile|ZFMK-TIS-2501452|Hesse
GBSBT016-18|Neobisium simile|ZFMK-TIS-2501451|Rhineland-Palatinate
GBSBT020-18|Neobisium simile|ZFMK-TIS-2501455|Hesse
GBPSS246-21|Dinocheirus panzeri|ZFMK-TIS-2627371|Hesse
GBPSS235-20|Dinocheirus panzeri|ZFMK-TIS-2586618|Hesse
GBPSS234-20|Dinocheirus panzeri|ZFMK-TIS-2586617|Hesse
GBPSS208-20|Dinocheirus panzeri|ZFMK-TIS-2586574|Brandenburg
GBPSS207-20|Dinocheirus panzeri|ZFMK-TIS-2586573|Brandenburg
GBPSS130-20|Dinocheirus panzeri|ZFMK-TIS-2568719|Rhineland-Palatinate
GBPSS036-20|Dinocheirus panzeri|ZFMK-TIS-2553318|Mecklenburg-Vorpommern
GBPSS196-20|Dinocheirus panzeri|ZFMK-TIS-2586551|Lower Saxony
GBPSS195-20|Dinocheirus panzeri|ZFMK-TIS-2586550|Lower Saxony
GBPSS236-20|Dinocheirus panzeri|ZFMK-TIS-2586619|Hesse
GBPSS188-20|Dinocheirus panzeri|ZFMK-TIS-2579795|Styria
GBPSS187-20|Dinocheirus panzeri|ZFMK-TIS-2579794|Styria
GBPSS035-20|Dinocheirus panzeri|ZFMK-TIS-2553317|Mecklenburg-Vorpommern
GBPSS233-20|Dinocheirus panzeri|ZFMK-TIS-2586607|Mecklenburg-Vorpommern
FBPSS053-16|Chernes nigrimanus|ZSM-BC-27399-E05|Bavaria
FBPSS071-16|Chernes nigrimanus|ZSM-BC-27399-F11|Bavaria
GBPSS186-20|Chernes nigrimanus|ZFMK-TIS-2579786|Styria
FBPSS055-16|Chernes nigrimanus|ZSM-BC-27399-E07|Bavaria
GBPSS185-20|Chernes nigrimanus|ZFMK-TIS-2579785|Styria
BSOIL304-18|Chernes nigrimanus|GBOL19949|Bavaria
FBPSS084-16|Chernes cimicoides|ZSM-BC-27399-G12|Bavaria
FBPSS072-16|Chernes cimicoides|ZSM-BC-27399-F12|Bavaria
FBPSS054-16|Chernes cimicoides|ZSM-BC-27399-E06|Bavaria
FBPSS051-16|Chernes cimicoides|ZSM-BC-27399-E03|Bavaria
FBPSS042-16|Chernes cimicoides|ZSM-BC-27399-D06|Bavaria
FBPSS040-16|Chernes cimicoides|ZSM-BC-27399-D04|Bavaria
FBPSS025-16|Chernes cimicoides|ZSM-BC-27399-C01|Bavaria
GBPSS124-20|Chernes cimicoides|ZFMK-TIS-2568703|Rhineland-Palatinate
GBPSS083-20|Chernes cimicoides|ZFMK-TIS-2555949|Mecklenburg-Vorpommern
GBPSS081-20|Chernes cimicoides|ZFMK-TIS-2555931|Mecklenburg-Vorpommern
GBPSS080-20|Chernes cimicoides|ZFMK-TIS-2555927|Mecklenburg-Vorpommern
GBPSS072-20|Chernes cimicoides|ZFMK-TIS-2555861|Mecklenburg-Vorpommern
GBPSS067-20|Chernes cimicoides|ZFMK-TIS-2555825|Mecklenburg-Vorpommern
GBPSS055-20|Chernes cimicoides|ZFMK-TIS-2555771|Mecklenburg-Vorpommern
GBPSS054-20|Chernes cimicoides|ZFMK-TIS-2555767|Mecklenburg-Vorpommern
GBPSS034-20|Chernes cimicoides|ZFMK-TIS-2553310|Mecklenburg-Vorpommern
GBPSS037-20|Chernes cimicoides|ZFMK-TIS-2553326|Mecklenburg-Vorpommern
FBPSS036-16|Chernes cimicoides|ZSM-BC-27399-C12|Bavaria
FBPSS035-16|Chernes cimicoides|ZSM-BC-27399-C11|Bavaria
FBPSS032-16|Chernes cimicoides|ZSM-BC-27399-C08|Bavaria
GBPSS075-20|Chernes cimicoides|ZFMK-TIS-2555870|Mecklenburg-Vorpommern
GBPSS062-20|Chernes cimicoides|ZFMK-TIS-2555805|Mecklenburg-Vorpommern
GBPSS203-20|Chernes cimicoides|ZFMK-TIS-2586566|Brandenburg
GBPSS141-20|Chernes cimicoides|ZFMK-TIS-2568744|Hesse
GBPSS104-20|Chernes cimicoides|ZFMK-TIS-2568659|Mecklenburg-Vorpommern
GBPSS103-20|Chernes cimicoides|ZFMK-TIS-2568658|Mecklenburg-Vorpommern
GBPSS082-20|Chernes cimicoides|ZFMK-TIS-2555945|Mecklenburg-Vorpommern
GBPSS079-20|Chernes cimicoides|ZFMK-TIS-2555894|Mecklenburg-Vorpommern
GBPSS078-20|Chernes cimicoides|ZFMK-TIS-2555891|Mecklenburg-Vorpommern
GBPSS077-20|Chernes cimicoides|ZFMK-TIS-2555888|Mecklenburg-Vorpommern
GBPSS076-20|Chernes cimicoides|ZFMK-TIS-2555885|Mecklenburg-Vorpommern
GBPSS066-20|Chernes cimicoides|ZFMK-TIS-2555823|Mecklenburg-Vorpommern
GBPSS065-20|Chernes cimicoides|ZFMK-TIS-2555819|Mecklenburg-Vorpommern
GBPSS058-20|Chernes cimicoides|ZFMK-TIS-2555791|Mecklenburg-Vorpommern
GBPSS056-20|Chernes cimicoides|ZFMK-TIS-2555775|Mecklenburg-Vorpommern
GBPSS204-20|Chernes cimicoides|ZFMK-TIS-2586567|Brandenburg
FBPSS029-16|Chernes cimicoides|ZSM-BC-27399-C05|Bavaria
BSOIL192-18|Chernes cimicoides|GBOL19837|Bavaria
BSOIL308-18|Chernes cimicoides|GBOL19953|Bavaria
GBPSS060-20|Chernes cimicoides|ZFMK-TIS-2555799|Mecklenburg-Vorpommern
BSOIL299-18|Chernes cimicoides|GBOL19944|Bavaria
GBPSS105-20|Chernes cimicoides|ZFMK-TIS-2568660|Mecklenburg-Vorpommern
FBPSS070-16|Chernes cimicoides|ZSM-BC-27399-F10|Bavaria
GMGMJ1489-14|Chernes cimicoides|BIOUG17099-E09|Rhineland-Palatinate
GBPSS073-20|Chernes cimicoides|ZFMK-TIS-2555864|Mecklenburg-Vorpommern
GBPSS184-20|Chernes cimicoides|ZFMK-TIS-2579784|Styria
GBPSS183-20|Chernes cimicoides|ZFMK-TIS-2579783|Styria
FBPSS038-16|Chernes hahnii|ZSM-BC-27399-D02|Bavaria
GBPSS165-20|Chernes hahnii|ZFMK-TIS-2576426|Saxony-Anhalt
GBPSS045-20|Chernes hahnii|ZFMK-TIS-2553344|Saxony
GBPSS245-20|Chernes hahnii|ZFMK-TIS-2615708|Saxony
GBPSS210-20|Chernes hahnii|ZFMK-TIS-2586579|Saxony
GBPSS198-20|Chernes hahnii|ZFMK-TIS-2586557|Lower Saxony
GBPSS044-20|Chernes hahnii|ZFMK-TIS-2553343|Saxony
GBPSS199-20|Chernes hahnii|ZFMK-TIS-2586558|Lower Saxony
GBPSS155-20|Dendrochernes cyrneus|ZFMK-TIS-2568779|Rhineland-Palatinate
GBPSS153-20|Dendrochernes cyrneus|ZFMK-TIS-2568777|Rhineland-Palatinate
GBPSS092-20|Dendrochernes cyrneus|ZFMK-TIS-2568637|Rhineland-Palatinate
GBPSS150-20|Dendrochernes cyrneus|ZFMK-TIS-2568759|Rhineland-Palatinate
GBPSS145-20|Dendrochernes cyrneus|ZFMK-TIS-2568748|Rhineland-Palatinate
GBPSS144-20|Dendrochernes cyrneus|ZFMK-TIS-2568747|Rhineland-Palatinate
GBPSS154-20|Dendrochernes cyrneus|ZFMK-TIS-2568778|Rhineland-Palatinate
GBPSS138-20|Dendrochernes cyrneus|ZFMK-TIS-2568740|Rhineland-Palatinate
GBPSS136-20|Dendrochernes cyrneus|ZFMK-TIS-2568735|Rhineland-Palatinate
GBPSS122-20|Dendrochernes cyrneus|ZFMK-TIS-2568701|Rhineland-Palatinate
GBPSS094-20|Dendrochernes cyrneus|ZFMK-TIS-2568640|Rhineland-Palatinate
GBPSS135-20|Dendrochernes cyrneus|ZFMK-TIS-2568734|Rhineland-Palatinate

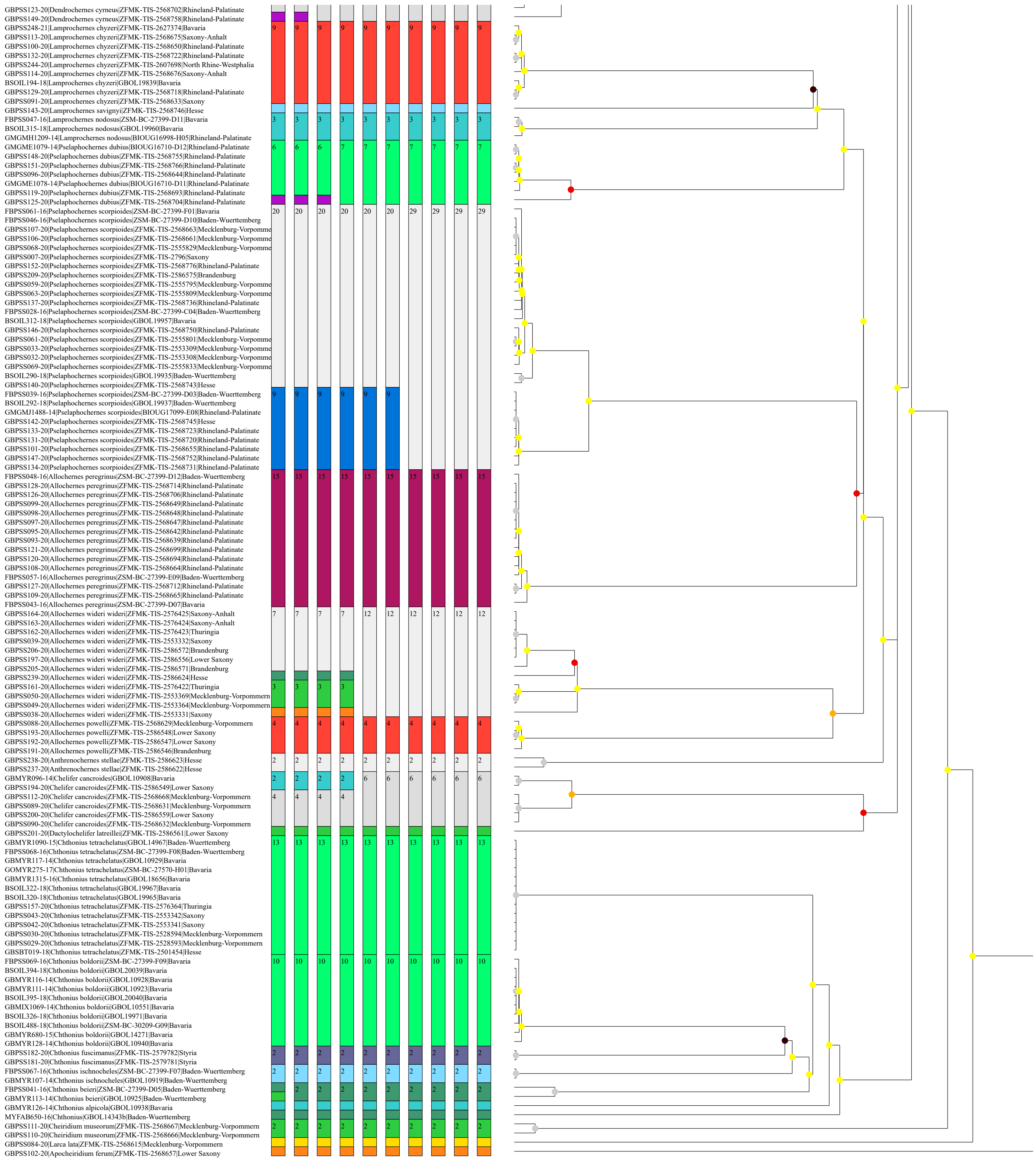
GBPSS123-20|Dendrochernes cyrneus|ZFMK-TIS-2568702|Rhineland-Palatinate
GBPSS149-20|Dendrochernes cyrneus|ZFMK-TIS-2568758|Rhineland-Palatinate
GBPSS248-21|Lamprochernes chyzeri|ZFMK-TIS-2627374|Bavaria
GBPSS113-20|Lamprochernes chyzeri|ZFMK-TIS-2568675|Saxony-Anhalt
GBPSS100-20|Lamprochernes chyzeri|ZFMK-TIS-2568650|Rhineland-Palatinate
GBPSS132-20|Lamprochernes chyzeri|ZFMK-TIS-2568722|Rhineland-Palatinate
GBPSS244-20|Lamprochernes chyzeri|ZFMK-TIS-2607698|North Rhine-Westphalia
GBPSS114-20|Lamprochernes chyzeri|ZFMK-TIS-2568676|Saxony-Anhalt
BSOIL194-18|Lamprochernes chyzeri|GBOL19839|Bavaria
GBPSS129-20|Lamprochernes chyzeri|ZFMK-TIS-2568718|Rhineland-Palatinate
GBPSS091-20|Lamprochernes chyzeri|ZFMK-TIS-2568633|Saxony
GBPSS143-20|Lamprochernes savignyi|ZFMK-TIS-2568746|Hesse
FBPSS047-16|Lamprochernes nodosus|ZSM-BC-27399-D11|Bavaria
BSOIL315-18|Lamprochernes nodosus|GBOL19960|Bavaria
GMGMH1209-14|Lamprochernes nodosus|BIOUG16998-H05|Rhineland-Palatinate
GMGME1079-14|Pselaphochernes dubius|BIOUG16710-D12|Rhineland-Palatinate
GBPSS148-20|Pselaphochernes dubius|ZFMK-TIS-2568755|Rhineland-Palatinate
GBPSS151-20|Pselaphochernes dubius|ZFMK-TIS-2568766|Rhineland-Palatinate
GBPSS096-20|Pselaphochernes dubius|ZFMK-TIS-2568644|Rhineland-Palatinate
GMGME1078-14|Pselaphochernes dubius|BIOUG16710-D11|Rhineland-Palatinate
GBPSS119-20|Pselaphochernes dubius|ZFMK-TIS-2568693|Rhineland-Palatinate
GBPSS125-20|Pselaphochernes dubius|ZFMK-TIS-2568704|Rhineland-Palatinate
FBPSS061-16|Pselaphochernes scorpioides|ZSM-BC-27399-F01|Bavaria
FBPSS046-16|Pselaphochernes scorpioides|ZSM-BC-27399-D10|Baden-Wuerttemberg
GBPSS107-20|Pselaphochernes scorpioides|ZFMK-TIS-2568663|Mecklenburg-Vorpomme
GBPSS106-20|Pselaphochernes scorpioides|ZFMK-TIS-2568661|Mecklenburg-Vorpomme
GBPSS068-20|Pselaphochernes scorpioides|ZFMK-TIS-2555829|Mecklenburg-Vorpomme
GBPSS007-20|Pselaphochernes scorpioides|ZFMK-TIS-2796|Saxony
GBPSS152-20|Pselaphochernes scorpioides|ZFMK-TIS-2568776|Rhineland-Palatinate
GBPSS209-20|Pselaphochernes scorpioides|ZFMK-TIS-2586575|Brandenburg
GBPSS059-20|Pselaphochernes scorpioides|ZFMK-TIS-2555795|Mecklenburg-Vorpomme
GBPSS063-20|Pselaphochernes scorpioides|ZFMK-TIS-2555809|Mecklenburg-Vorpomme
GBPSS137-20|Pselaphochernes scorpioides|ZFMK-TIS-2568736|Rhineland-Palatinate
FBPSS028-16|Pselaphochernes scorpioides|ZSM-BC-27399-C04|Baden-Wuerttemberg
BSOIL312-18|Pselaphochernes scorpioides|GBOL19957|Bavaria
GBPSS146-20|Pselaphochernes scorpioides|ZFMK-TIS-2568750|Rhineland-Palatinate
GBPSS061-20|Pselaphochernes scorpioides|ZFMK-TIS-2555801|Mecklenburg-Vorpomme
GBPSS033-20|Pselaphochernes scorpioides|ZFMK-TIS-2553309|Mecklenburg-Vorpomme
GBPSS032-20|Pselaphochernes scorpioides|ZFMK-TIS-2553308|Mecklenburg-Vorpomme
GBPSS069-20|Pselaphochernes scorpioides|ZFMK-TIS-2555833|Mecklenburg-Vorpomme
BSOIL290-18|Pselaphochernes scorpioides|GBOL19935|Baden-Wuerttemberg
GBPSS140-20|Pselaphochernes scorpioides|ZFMK-TIS-2568743|Hesse
FBPSS039-16|Pselaphochernes scorpioides|ZSM-BC-27399-D03|Baden-Wuerttemberg
BSOIL292-18|Pselaphochernes scorpioides|GBOL19937|Baden-Wuerttemberg
GMGMJ1488-14|Pselaphochernes scorpioides|BIOUG17099-E08|Rhineland-Palatinate
GBPSS142-20|Pselaphochernes scorpioides|ZFMK-TIS-2568745|Hesse
GBPSS133-20|Pselaphochernes scorpioides|ZFMK-TIS-2568723|Rhineland-Palatinate
GBPSS131-20|Pselaphochernes scorpioides|ZFMK-TIS-2568720|Rhineland-Palatinate
GBPSS101-20|Pselaphochernes scorpioides|ZFMK-TIS-2568655|Rhineland-Palatinate
GBPSS147-20|Pselaphochernes scorpioides|ZFMK-TIS-2568752|Rhineland-Palatinate
GBPSS134-20|Pselaphochernes scorpioides|ZFMK-TIS-2568731|Rhineland-Palatinate
FBPSS048-16|Allochernes peregrinus|ZSM-BC-27399-D12|Baden-Wuerttemberg
GBPSS128-20|Allochernes peregrinus|ZFMK-TIS-2568714|Rhineland-Palatinate
GBPSS126-20|Allochernes peregrinus|ZFMK-TIS-2568706|Rhineland-Palatinate
GBPSS099-20|Allochernes peregrinus|ZFMK-TIS-2568649|Rhineland-Palatinate
GBPSS098-20|Allochernes peregrinus|ZFMK-TIS-2568648|Rhineland-Palatinate
GBPSS097-20|Allochernes peregrinus|ZFMK-TIS-2568647|Rhineland-Palatinate
GBPSS095-20|Allochernes peregrinus|ZFMK-TIS-2568642|Rhineland-Palatinate
GBPSS093-20|Allochernes peregrinus|ZFMK-TIS-2568639|Rhineland-Palatinate
GBPSS121-20|Allochernes peregrinus|ZFMK-TIS-2568699|Rhineland-Palatinate
GBPSS120-20|Allochernes peregrinus|ZFMK-TIS-2568694|Rhineland-Palatinate
GBPSS108-20|Allochernes peregrinus|ZFMK-TIS-2568664|Rhineland-Palatinate
FBPSS057-16|Allochernes peregrinus|ZSM-BC-27399-E09|Baden-Wuerttemberg
GBPSS127-20|Allochernes peregrinus|ZFMK-TIS-2568712|Rhineland-Palatinate
GBPSS109-20|Allochernes peregrinus|ZFMK-TIS-2568665|Rhineland-Palatinate
FBPSS043-16|Allochernes peregrinus|ZSM-BC-27399-D07|Bavaria
GBPSS164-20|Allochernes wideri wideri|ZFMK-TIS-2576425|Saxony-Anhalt
GBPSS163-20|Allochernes wideri wideri|ZFMK-TIS-2576424|Saxony-Anhalt
GBPSS162-20|Allochernes wideri wideri|ZFMK-TIS-2576423|Thuringia
GBPSS039-20|Allochernes wideri wideri|ZFMK-TIS-2553332|Saxony
GBPSS206-20|Allochernes wideri wideri|ZFMK-TIS-2586572|Brandenburg
GBPSS197-20|Allochernes wideri wideri|ZFMK-TIS-2586556|Lower Saxony
GBPSS205-20|Allochernes wideri wideri|ZFMK-TIS-2586571|Brandenburg
GBPSS239-20|Allochernes wideri wideri|ZFMK-TIS-2586624|Hesse
GBPSS161-20|Allochernes wideri wideri|ZFMK-TIS-2576422|Thuringia
GBPSS050-20|Allochernes wideri wideri|ZFMK-TIS-2553369|Mecklenburg-Vorpommern
GBPSS049-20|Allochernes wideri wideri|ZFMK-TIS-2553364|Mecklenburg-Vorpommern
GBPSS038-20|Allochernes wideri wideri|ZFMK-TIS-2553331|Saxony
GBPSS088-20|Allochernes powelli|ZFMK-TIS-2568629|Mecklenburg-Vorpommern
GBPSS193-20|Allochernes powelli|ZFMK-TIS-2586548|Lower Saxony
GBPSS192-20|Allochernes powelli|ZFMK-TIS-2586547|Lower Saxony
GBPSS191-20|Allochernes powelli|ZFMK-TIS-2586546|Brandenburg
GBPSS238-20|Anthrenochernes stellae|ZFMK-TIS-2586623|Hesse
GBPSS237-20|Anthrenochernes stellae|ZFMK-TIS-2586622|Hesse
GBMYR096-14|Chelifer cancroides|GBOL10908|Bavaria
GBPSS194-20|Chelifer cancroides|ZFMK-TIS-2586549|Lower Saxony
GBPSS112-20|Chelifer cancroides|ZFMK-TIS-2568668|Mecklenburg-Vorpommern
GBPSS089-20|Chelifer cancroides|ZFMK-TIS-2568631|Mecklenburg-Vorpommern
GBPSS200-20|Chelifer cancroides|ZFMK-TIS-2586559|Lower Saxony
GBPSS090-20|Chelifer cancroides|ZFMK-TIS-2568632|Mecklenburg-Vorpommern
GBPSS201-20|Dactylochelifer latreillei|ZFMK-TIS-2586561|Lower Saxony
GBMYR1090-15|Chthonius tetrachelatus|GBOL14967|Baden-Wuerttemberg
FBPSS068-16|Chthonius tetrachelatus|ZSM-BC-27399-F08|Baden-Wuerttemberg
GBMYR117-14|Chthonius tetrachelatus|GBOL10929|Bavaria
GOMYR275-17|Chthonius tetrachelatus|ZSM-BC-27570-H01|Bavaria
GBMYR1315-16|Chthonius tetrachelatus|GBOL18656|Bavaria
BSOIL322-18|Chthonius tetrachelatus|GBOL19967|Bavaria
BSOIL320-18|Chthonius tetrachelatus|GBOL19965|Bavaria
GBPSS157-20|Chthonius tetrachelatus|ZFMK-TIS-2576364|Thuringia
GBPSS043-20|Chthonius tetrachelatus|ZFMK-TIS-2553342|Saxony
GBPSS042-20|Chthonius tetrachelatus|ZFMK-TIS-2553341|Saxony
GBPSS030-20|Chthonius tetrachelatus|ZFMK-TIS-2528594|Mecklenburg-Vorpommern
GBPSS029-20|Chthonius tetrachelatus|ZFMK-TIS-2528593|Mecklenburg-Vorpommern
GBSBT019-18|Chthonius tetrachelatus|ZFMK-TIS-2501454|Hesse
FBPSS069-16|Chthonius boldorii|ZSM-BC-27399-F09|Bavaria
BSOIL394-18|Chthonius boldorii|GBOL20039|Bavaria
GBMYR116-14|Chthonius boldorii|GBOL10928|Bavaria
GBMYR111-14|Chthonius boldorii|GBOL10923|Bavaria
BSOIL395-18|Chthonius boldorii|GBOL20040|Bavaria
GBMIX1069-14|Chthonius boldorii|GBOL10551|Bavaria
BSOIL326-18|Chthonius boldorii|GBOL19971|Bavaria
BSOIL488-18|Chthonius boldorii|ZSM-BC-30209-G09|Bavaria
GBMYR680-15|Chthonius boldorii|GBOL14271|Bavaria
GBMYR128-14|Chthonius boldorii|GBOL10940|Bavaria
GBPSS182-20|Chthonius fuscimanus|ZFMK-TIS-2579782|Styria
GBPSS181-20|Chthonius fuscimanus|ZFMK-TIS-2579781|Styria
FBPSS067-16|Chthonius ischnocheles|ZSM-BC-27399-F07|Baden-Wuerttemberg
GBMYR107-14|Chthonius ischnocheles|GBOL10919|Baden-Wuerttemberg
FBPSS041-16|Chthonius beieri|ZSM-BC-27399-D05|Baden-Wuerttemberg
GBMYR113-14|Chthonius beieri|GBOL10925|Baden-Wuerttemberg
GBMYR126-14|Chthonius alpicola|GBOL10938|Bavaria
MYFAB650-16|Chthonius|GBOL14343b|Baden-Wuerttemberg
GBPSS111-20|Cheiridium museorum|ZFMK-TIS-2568667|Mecklenburg-Vorpommern
GBPSS110-20|Cheiridium museorum|ZFMK-TIS-2568666|Mecklenburg-Vorpommern
GBPSS084-20|Larca lata|ZFMK-TIS-2568615|Mecklenburg-Vorpommern
GBPSS102-20|Apocheiridium ferum|ZFMK-TIS-2568657|Lower Saxony
